# Palmitoylation of PSD‐95 Orchestrates Learning‐Dependent Metaplasticity in the Amygdala and Fear Memory

**DOI:** 10.1002/advs.76302

**Published:** 2026-07-06

**Authors:** Zu‐Cheng Shen, Yu‐Xuan Weng, Zhi‐Xuan Xia, Yue‐Ling Zhao, Qian‐Qian Ding, Si‐Ying Wang, Ting Cao, Xuan‐Ying Chen, Wu‐Cheng Tao, Shen Lin, Zhou Chen, Yi‐Xiao Luo

**Affiliations:** ^1^ Department of Pharmacology School of Pharmacy Fujian Medical University Fuzhou China; ^2^ Key Laboratory of Brain Aging and Neurodegenerative Diseases Fujian Medical University Fuzhou China; ^3^ School of Basic Medicine and Life Sciences Key Laboratory of Tropical Translational Medicine of Ministry of Education Hainan Medical University Haikou China; ^4^ Department of Stomatology Union Hospital Fujian Medical University Fuzhou China; ^5^ Fujian Provincial Institutes of Brain Disorders and Brain Sciences First Affiliated Hospital Fujian Medical University Fuzhou China; ^6^ Department of Clinical Pharmacy and Pharmacy Administration School of Pharmacy Fujian Medical University Fuzhou China; ^7^ Beijing Key Laboratory of Intelligent Drug Research and Development for Mental Disorders National Clinical Research Center for Mental Disorders National Center for Mental Disorders Beijing Anding Hospital Capital Medical University Beijing China; ^8^ School of Pharmaceutical Sciences Hunan Normal University Changsha China

**Keywords:** fear memory, lateral amygdala, Palmitoylation, PSD‐95

## Abstract

Palmitoylation has been implicated in learning and memory processes, yet its precise role and underlying mechanisms remain poorly understood. Here, we report a novel role of palmitoylation in regulating amygdalar synaptic plasticity and fear memory, involving the regulation of palmitate cycling on PSD‐95. Using a series of multi‐methodological experiments, we demonstrated that conditioned fear learning impairs amygdalar metaplasticity—an effect not observed in the hippocampus. Furthermore, PSD‐95 recruits LTP‐like mechanisms at lateral amygdala (LA) synapses, and its palmitoylation is critically involved in fear memory expression. Notably, PSD‐95 palmitoylation contributes to the occlusion of LA‐LTP following fear conditioning. Mechanistically, DHHC2 drives PSD‐95 palmitoylation and modulates LA synaptic plasticity following fear conditioning, whereas PICK1 facilitates the activity‐dependent trafficking of DHHC2 to PSD‐95 at synapses. Collectively, these findings highlight the PICK1‐DHHC2‐PSD‐95 pathway as a critical regulator of learning‐dependent metaplasticity in the LA, which occludes further HFS‐induced LTP while promoting fear memory expression.

## Graphical abstract

This study reveals that fear learning specifically triggers PICK1/DHHC2‐dependent palmitoylation of PSD‐95 in the lateral amygdala. Fear conditioning induces the dissociation of DHHC5 from PSD‐95 and the association of PSD‐95 with DHHC2. DHHC2‐mediated palmitoylation of PSD‐95 is required for synaptic transmission and underlies fear learning–induced LTP impairment, with PICK1 mediating activity‐dependent trafficking of DHHC2 to PSD‐95.

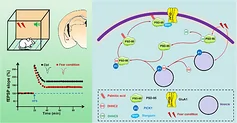

## 1 Introduction

Fear‐related anxiety disorders, such as social anxiety disorder, post‐traumatic stress disorder (PTSD), and panic disorder, are among the most prevalent psychiatric conditions worldwide [1, 2, 3]. While PTSD is characterized by diverse molecular phenotypes, insufficient understanding of its pathophysiology at the cellular and molecular levels remains a key barrier to the development of novel therapeutic interventions. Although the precise mechanisms underlying fear memory remain incompletely elucidated, accumulating evidence indicates that fear memory expression and consolidation are strongly linked to hyperexcitability in the basolateral amygdala (BLA) [4, 5].

Fear memory formation relies on experience‐dependent synaptic plasticity in the BLA—a core hub for the acquisition, storage, and expression of conditioned fear—comprising approximately 90% glutamatergic pyramidal neurons [6, 7]. BLA neurons exhibit hyperexcitability following fear conditioning [8], and circumstantial evidence indicates that enhanced long‐term potentiation (LTP) in the BLA is associated with fear learning [9, 10]. Fear conditioning strengthens amygdalar synaptic transmission, a phenomenon that resembles LTP [11]; notably, antagonists of N‐methyl‐D‐aspartate receptors (NMDARs) block the induction of both amygdalar LTP and fear learning [12]. However, the BLA may also undergo persistent modifications that reduce its plasticity after fear conditioning. The BLA receives convergent inputs from the prefrontal cortex, thalamus, and other limbic structures implicated in fear and fear conditioning processes [13, 14, 15]. NMDAR‐dependent LTP in the cortico‐BLA pathway plays a critical role in auditory fear conditioning: fear‐induced persistent potentiation of glutamatergic synaptic transmission occludes further LTP induction, which may impair the neurons’ residual capacity to store new information [16]. Nevertheless, the precise synaptic modifications that underlie cortico‐amygdalar LTP and the acquisition of fear memory remain unexplored.

Palmitoylation, a reversible lipid modification, refers to the post‐translational addition of saturated 16‐carbon palmitic acid to cysteine residues via the formation of a labile thioester linkage [17, 18]. Intracellular palmitoylation reactions are catalyzed by a family of proteins containing the aspartate‐histidine‐histidine‐cysteine (DHHC) domain, which are now recognized as palmitoyl acyltransferases [19]. Palmitoylation reversibly regulates a variety of proteins, including neurotransmitter receptors, synaptic scaffolding proteins, and secreted signaling molecules, and modulates their stability, trafficking, subcellular localization, and signaling activity [20, 21]. Importantly, palmitoylation has been implicated in neuropsychiatric disorders such as schizophrenia and Huntington's disease [22, 23]. For example, genetic knockout of ZDHHC8—an enzyme that mediates the palmitoylation of postsynaptic density protein 95 (PSD‐95), a molecule involved in synaptic plasticity, learning, and memory—results in deficits in prepulse inhibition and reduced exploratory activity in ZDHHC8‐deficient mice [24]. However, the regulatory mechanisms by which palmitoylation modulates learning, particularly the acquisition of fear memory, remain unexplored.

In the present study, we first investigated whether palmitoylation is essential for synaptic transmission at the synaptic network level; we then sought to determine whether palmitoylation is involved in regulating LTP impairment in the LA subsequent to fear memory acquisition. We also explored the regulatory mechanisms underlying the crosstalk between palmitoylation cycling and synaptic plasticity in the LA, and verified the role of protein interacting with C‐Kinase 1 (PICK1)‐mediated DHHC2 trafficking in fear memory formation. Collectively, this study identifies a novel molecular mechanism underlying abnormal palmitoylation‐induced LTP occlusion and synaptic plasticity impairment in the LA following fear conditioning, and offers new insights into the development of therapeutic interventions for fear‐related disorders such as PTSD.

## 2 Results

### 2.1 Fear Conditioning Compromises Metaplasticity in the Amygdala

**FIGURE 1:**
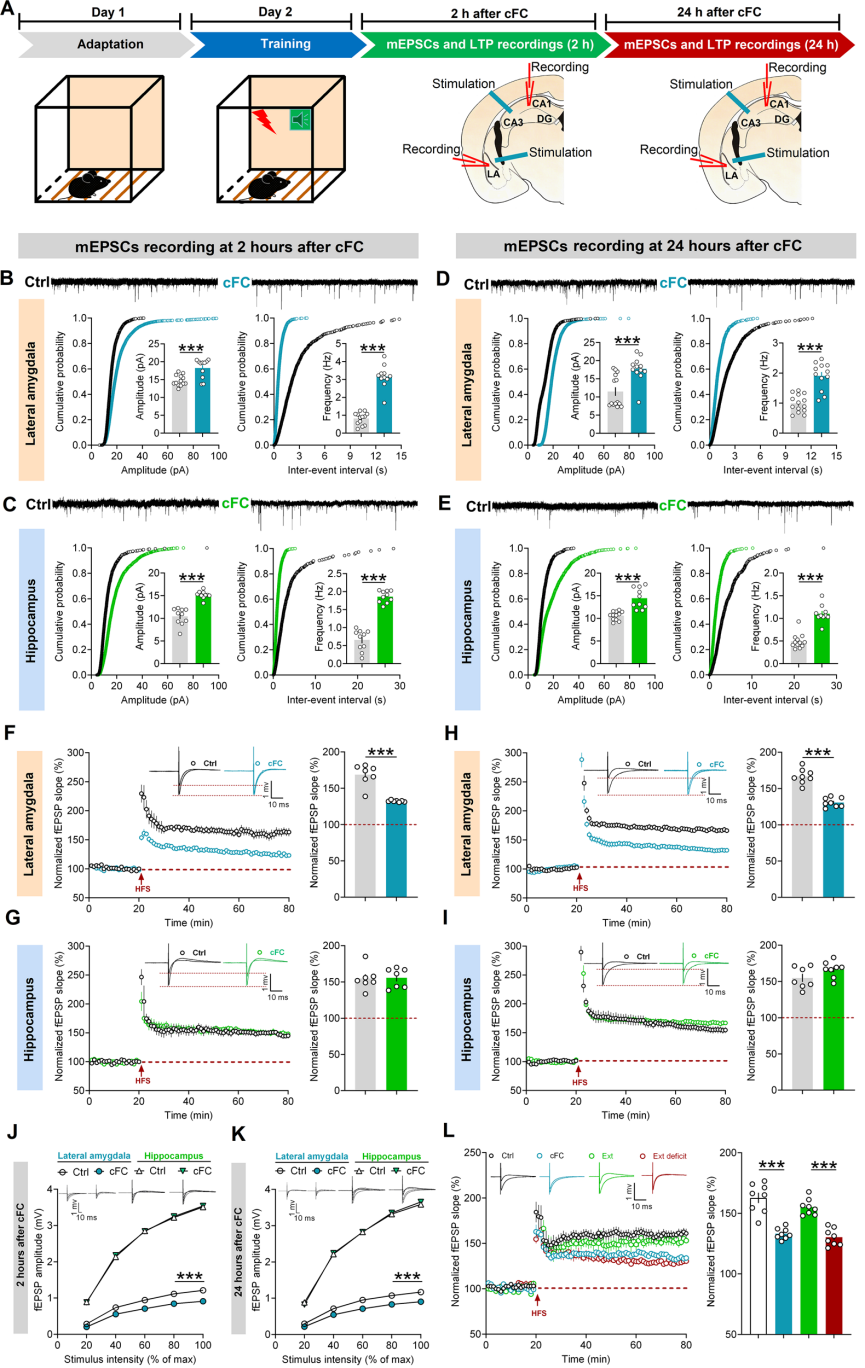
Fear learning impairs metaplasticity in the lateral amygdala. (A) Schematic timeline of cue fear conditioning (cFC) paradigm and subsequent electrophysiological recordings workflow. (B,C) Representative mEPSCs traces (top), along with cumulative probability distribution, average frequency and amplitude (bottom), recorded in the LA (B) and hippocampus (C) at 2 h following cFC (n = 11–13 cells from 5 rats for B, n = 10 cells from 5 rats for C). (D,E) Representative mEPSCs traces (top) and cumulative probability distributions, average frequency and amplitude (bottom), recorded in the LA (D) and hippocampus (E) at 24 h following cFC (n = 12–14 cells from 5 rats for D, n = 10–12 cells from 5 rats for E). (F–I) Time course of LTP recorded in the LA and hippocampal slices at 2 h (F and G) and 24 h (H,I) following cFC. Representative fEPSP traces from Ctrl and cFC groups are inset in the upper right (scale bars: 1 mV/10 ms). Right‐side histograms show LTP magnitude averaged from the final 15 min of recordings (n = 7 slices from 4 rats for F, n = 7 slices from 4 rats for G, n = 8 slices from 4 rats for H, n = 7–8 slices from 4 rats for I). (J,K) Input‐output curves of fEPSP amplitude in the LA at 2 h (J) and 24 h (K) following cFC (n = 6 rats per group). (L) Time‐course of HFS‐induced LTP in the LA from Ctrl, cFC, Extinction, and Extinction Deficit groups. Representative fEPSP traces from the four groups are inset in the upper right (scale bars: 1 mV/10 ms). The right‐side histogram shows LTP magnitude, averaged from the final 15 min of recordings (n = 8 slices from 4 rats). Data are represented as mean ± SEM. ^***^ *p* < 0.001. Statistical analyses were performed using unpaired two‐tailed Student's *t*‐test (B to K) and one‐way ANOVA followed by Tukey’ s post hoc test (L).

Long‐term potentiation (LTP) represents a core manifestation of synaptic plasticity and a key neural substrate for the encoding and storage of fear memory [25, 26, 27], whereas the amygdala—particularly the lateral amygdala (LA)— serves as the pivotal brain region governing fear memory processing [28]. Accumulating evidence confirms that fear learning specifically induces LTP impairment in the amygdala [29, 30]; however, its underlying neurobiological mechanisms remain poorly understood. To characterize and dissect the impact of fear learning on amygdala synaptic plasticity, we selected another brain region associated with learning and memory—the hippocampus [31], to compare with the amygdala, and performed cue fear conditioning (cFC) in rats, followed by recordings of miniature excitatory postsynaptic currents (mEPSCs) and LTP in the LA and hippocampus at 2 h and 24 h post‐training (Figure 1A). Following validation of successful cFC model establishment (Figure S1), we performed electrophysiological recordings and found that fear conditioning alters mEPSCs properties in the LA and hippocampus. At 2 h post‐cFC, compared with the control (Ctrl) group, the cFC group exhibited a marked increase in both mEPSCs amplitude and frequency in the LA (amplitude: Ctrl 14.71 ± 0.41 pA vs. cFC 18.23 ± 0.80 pA, frequency: Ctrl 0.81 ± 0.09 Hz vs. cFC 3.16 ± 0.19 Hz, Figure 1B). Similar results were also observed in the hippocampus (amplitude: Ctrl 10.42 ± 0.58 pA vs. cFC 15.16 ± 0.26 pA, frequency: Ctrl 0.66 ± 0.09 Hz vs. cFC 1.86 ± 0.05 Hz, Figure 1C). These bidirectional changes were sustained at 24 h post‐cFC mEPSCs amplitude (Ctrl 11.49 ± 1.17 pA vs. cFC 17.79 ± 1.01 pA) and frequency (Ctrl 0.99 ± 0.07 Hz vs. cFC 1.90 ± 0.13 Hz) remained upregulated in the LA region (Figure 1D), and hippocampal mEPSCs amplitude (Ctrl 10.06 ± 0.27 pA vs. cFC 14.45 ± 0.72 pA) and frequency (Ctrl 0.49 ± 0.04 Hz vs. cFC 1.1 ± 0.06 Hz) stayed elevated (Figure 1E).

Furthermore, at 2 h post‐cFC, LTP induction was significantly impaired in the LA of the cFC‐exposed rats, as evidenced by the reduced normalized EPSP slope relative to the Ctrl group (Ctrl 168.56 ± 6.06% vs. cFC 132.25 ± 0.51%, Figure 1F). Conversely, hippocampal LTP was largely preserved in the cFC group, with a slightly higher normalized EPSP slope compared with the Ctrl group (Ctrl 154.73 ± 6.34% vs. cFC 155.71 ± 5.50%, Figure 1G). Notably, this bidirectional regulatory pattern of LTP persisted at 24 h post‐cFC: LA LTP remained suppressed (Ctrl 166.87 ± 3.60% vs. cFC 130.78 ± 2.25%, Figure 1H), where hippocampal LTP stayed potentiated (Ctrl 154.97 ± 5.64% vs. cFC 166.67 ± 3.93%, Figure 1I). To further elucidate the metaplasticity changes in the LA and hippocampus following cFC, we assessed the input/output (I/O) curves in acute brain slices of these regions. At 2 h post‐cFC, the cFC group displayed blunted synaptic responsiveness (EPSP amplitude vs stimulus intensity) in the LA, whereas hippocampal synaptic responsiveness was significantly potentiated (Figure 1J). A comparable pattern of region‐specific metaplasticity was also detected at 24 h post‐cFC (Figure 1K). These results imply that, compared with the hippocampus region, fear conditioning may contribute to the development of pathological fear memories (e.g. PTSD) by disrupting amygdalar metaplasticity. To test this hypothesis, we subjected rats to fear conditioning followed by extinction training, and stratified the animals into four experimental groups: Ctrl, cFC, extinction (Ext), and extinction deficit (Ext deficit) groups. Subsequent LTP recordings verified that fear conditioning indeed induces LA LTP impairment. Importantly, LTP was fully restored in the Ext group, whereas LTP remained impaired in the Ext deficit group—accompanied by persistent fear memory (Figure 1L). Taken together, our findings further demonstrate that compromised amygdalar metaplasticity, manifested as LA LTP impairment, is critically involved in the pathogenesis of pathological fear memories.

### 2.2 PSD‐95 Recruits LTP‐Like Molecular Mechanisms at LA Synapses

**FIGURE 2:**
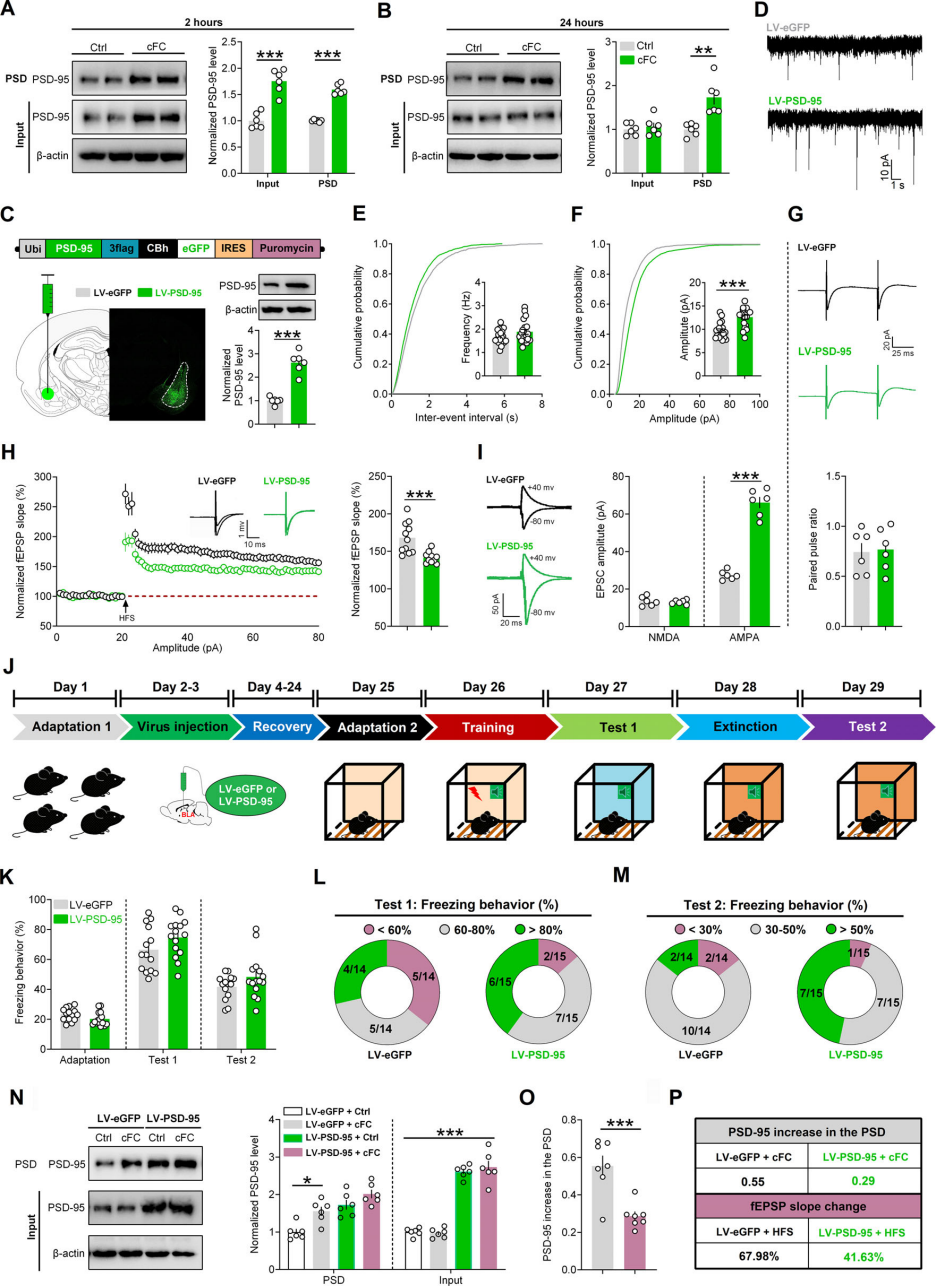
PSD‐95 engages L‐LTP‐like synaptic mechanisms at LA synapses. (A and B) Representative Western blots and quantitative analysis of PSD‐95 in total lysate (Input) and post‐synaptic density (PSD) fractions in LA tissue from Ctrl and cFC rats at 2 h (A) and 24 h (B) following cFC (n = 6 rats per group). (C) Schematic diagram of the LV‐PSD‐95 construct, representative stereotaxic injection coordinates, and immunofluorescence staining of coronal brain sections showing the expression of PSD‐95 in the LA. Below: Representative Western blots and quantification of PSD‐95 expression in LA tissues from LV‐eGFP and LV‐PSD‐95 groups (n = 6 rats per group). (D to F) Representative mEPSCs traces (D), and cumulative probability distributions, along with average frequency (E) and amplitude (F), recorded from LA neurons transduced with LV‐eGFP or LV‐PSD‐95 (n = 20 cells from 8 rats). (G) Representative paired‐pulse ratio (PPR) traces (top) and quantification (bottom)(n = 6 cells from 3 rats). (H) Time course of HFS‐induced LTP in the LA from LV‐eGFP or LV‐PSD‐95 groups. Representative fEPSP traces are inset in the upper right (scale bars: 1 mV/10 ms). The right‐side histogram shows LTP magnitude, averaged from the final 15 min of recordings (n = 10–11 slices from 5 rats). (I) Representative EPSC traces recorded at ‐80 mV (AMPAs currents) and +40 mV (NMDA currents)(scale bars: 50 pA/20 ms), with their respective quantification (n = 6 cells from 3 rats). (J) Schematic timeline of the experimental procedures. (K) Freezing behavior of rats injected with LV‐eGFP o LV‐PSD‐95 during cFC tests (n = 13–15 rats per group). (L and M) Distribution of the proportion of animals across different freezing levels in each group during Test 1 (L) and Test 2 (M) (n = 13–15 rats per group). (N) Representative Western blots (left) and quantitative analysis (right) of PSD‐95 expression in total tissue lysate (Input) and postsynaptic density (PSD) fractions from the LA of rats subjected to LV‐PSD‐95 (or LV‐eGFP control) injection and/or cFC (n = 6 rats per group). (O) Magnitude of cFC‐induced PSD‐95 increase in PSD fractions, comparing LV‐eGFP control and LV‐PSD‐95 groups (n = 7 rats per group). (P) Magnitude of PSD‐95 increase in PSD fractions and HFS‐induced fEPSP slope change in rats subjected to LV‐PSD‐95 overexpression (or LV‐eGFP control) and/or cFC. Data are represented as mean ± SEM. ^*^ *p* < 0.05, ^**^ *p* < 0.01, ^***^ *p* < 0.001. Statistical analyses were performed using one‐way ANOVA followed by Tukey’ s post hoc test (A–K, O) and unpaired two‐tailed Student's *t*‐test (N).

LTP is associated with increased quantal size and frequency of mEPSCs [32], while postsynaptic density protein 95 (PSD‐95) regulates synaptic strength in an activity‐dependent manner [33]. Notably, elevated PSD‐95 levels both mimic and occlude endogenous LTP and experience‐driven synaptic plasticity in vivo [34, 35]. Thus, we sought to determine whether PSD‐95 mediates the impairment of LA metaplasticity following fear conditioning. First, we assessed PSD‐95 expression and its enrichment in the postsynaptic density (PSD) fraction of LA region at 2 and 24 h post‐cFC. As shown in Figure 2A, PSD‐95 expression and its PSD fraction enrichment were significantly increased in the cFC group compared to controls at 2 h post‐cFC. In contrast, at 24 h post‐cFC, only a significant increase in PSD‐95 enrichment in the PSD fraction was detected in the LA region (Figure 2B), with no notable change in total PSD‐95 expression. These results indicate that cFC drives the specific recruitment of PSD‐95 to LA synaptic sites.

To further investigate the role of PSD‐95 in amygdala synaptic plasticity and fear memory regulation induced by cFC, we performed virus‐mediated PSD‐95 overexpression in the LA using a lentiviral vector (LV‐PSD‐95), with LV‐eGFP as the control (Figure S2). First, we validated the specificity and efficiency of LA‐targeted PSD‐95 overexpression: fluorescence imaging confirmed selective viral localization to the LA region, and immunoblotting analysis demonstrated that PSD‐95 protein levels in LA tissue were significantly elevated in the LV‐PSD‐95 group relative to the LV‐eGFP control group (Figure 2C). Next, we evaluated the impact of PSD‐95 overexpression on LA synaptic transmission. As expected, mEPSCs amplitude was significantly increased in the LV‐PSD‐95 group, with no significant alteration in mEPSCs frequency (Figure 2D–F). To evaluate presynaptic neurotransmitter release probability, we measured paired‐pulse facilitation (PPF), a well‐established indicator of presynaptic function [36]. No obvious difference in PPF was observed between LV‐eGFP and LV‐PSD‐95 groups (Figure 2G), suggesting that PSD‐95 overexpression does not affect presynaptic release. For LTP recordings, consistent with the LTP impairment induced by cFC, the normalized EPSP slope was significantly lower in the LV‐PSD‐95 group compared to the LV‐eGFP control group (Figure 2H). This was accompanied by a significant increased in the NMDA/AMPA receptor current ratio (Figure 2I), indicating that PSD‐95 enhances basal synaptic transmission by adding AMPARs to synapses.

To investigate the role of PSD‐95 in cFC‐induced fear memory, we conducted behavioral and molecular assays following the experimental timeline depicted in Figure 2J: rats underwent adaptation, lentiviral (LV‐eGFP/LV‐PSD‐95) injection targeting the LA region, recovery, cFC training, fear memory testing (Test 1), extinction training, and post‐extinction memory testing (Test 2). As shown by the results, freezing behavior did not differ significantly between the LV‐eGFP and LV‐PSD‐95 groups during the training phase. In Test 1, freezing behavior was slightly elevated in the LV‐PSD‐95 group relative to the LV‐eGFP group, while no marked difference in freezing was detected between two groups in Test 2 (Figure 2K). Distribution analysis of Test 1 freezing behavior further revealed a greater prevalence of high fear responses in the LV‐PSD‐95 group: ∼64% of LV‐eGFP rats exhibited freezing > 60%, while over 86% of LV‐PSD‐95 rats showed freezing > 60% (Figure 2L). This pattern indicates that PSD‐95 overexpression strengthens fear memory formation. In Test 2, LV‐eGFP rats displayed markedly reduced freezing (2/14 rats showed freezing > 50%), whereas LV‐PSD‐95 rats retained high freezing levels (7/15 rats with freezing > 50%, Figure 2M), demonstrating PSD‐95 enhances fear memory expression and impairs extinction. Notably, this behavioral profile, characterized by a high proportion of resistant fear responses and failed extinctio, closely parallels the natural “extinction‐deficit” phenotype, suggesting that excessive synaptic PSD‐95 may drive a structural stabilization that limits behavioral updating.

Next, we assessed PSD‐95 levels in the PSD fraction via immunoblotting. As shown in Figure 2N, cFC treatment significantly increased synaptic PSD‐95 enrichment in the LV‐eGFP control group, whereas no significant change in synaptic PSD‐95 levels was detected in the LV‐PSD‐95 overexpression group following cFC. To further characterize this effect, we compared the magnitude of cFC‐induced PSD‐95 increase in the PSD fraction across all four groups. As depicted in Figure 2O, PSD‐95 overexpression attenuated the amplitude of the cFC‐induced elevation in synaptic PSD‐95 enrichment, indicating that exogenous PSD‐95 may saturate synaptic compartments and limit further recruitment of endogenous PSD‐95 following cFC. We next quantified the relationship between the cFC‐induced increase in PSD‐95 (in the PSD fraction) and changes in the fEPSP slope. In LV‐eGFP+cFC rats, PSD‐95 in the PSD fraction increased by 0.55, accompanied by a 67.98% elevation in the fEPSP slope following HFS. In contrast, LV‐PSD‐95+cFC rats exhibited a 0.29 increase in synaptic PSD‐95, paired with only a 41.63% increase in the fEPSP slope (Figure 2P). Importantly, this reveals a quantitative correlation aligning with our metaplasticity model: the degree of synaptic PSD‐95 saturation correlates with the magnitude of LTP impairment. Additionally, we investigated the surface expression of glutamate receptors following PSD‐95 overexpression and cFC. Consistent with our previous findings, enhanced synaptic PSD‐95 enrichment did not alter the surface expression of NMDARs (Figure S3). Notably, a similar pattern to that of PSD‐95 was observed for the surface expression of GluA1 (Figure S4). Collectively, this negative correlation between synaptic PSD‐95 enrichment and LTP magnitude indicates that excessive accumulation of PSD‐95 at LA synapses is associated with impaired LTP.

### 2.3 PSD‐95 Palmitoylation is Required for Fear Memory Expression

Our above findings demonstrate that PSD‐95 modulates fear memory formation and associated synaptic plasticity in the LA by potentially targeting its enrichment in the PSD and the recruitment of AMPARs to the postsynaptic membrane. Notably, PSD‐95 localized within the synaptic PSD is highly palmitoylated [37], and this post‐translational modification promotes the synaptic localization and stability of PSD‐95 in the PSD [38]. In contrast, palmitoylation induces the internalization and trafficking of AMPARs to the Golgi apparatus [39]. Thus, we wondered whether PSD‐95 regulates fear memory expression and associated metaplasticity impairment in the LA through its palmitoylation. First, to rule out the possibility that the tone (conditional stimulus, CS) or foot shock (unconditional stimulus, US) alone exerts an effect on protein palmitoylation, we collected LA tissue samples 24 h after a single exposure to either CS or US, and carried out the acyl‐biotin exchange (ABE) assay. As shown in Figure S5A, neither CS nor US alone had a significant impact on the global palmitoylation level in the LA. In contrast, compared with control animals, fear‐conditioned rats exhibited a marked increase in global palmitoylation levels in the LA in the cFC model (Figure S5B). Collectively, these results suggest that protein palmitoylation may play a critical role in fear memory formation in the LA.

**FIGURE 3:**
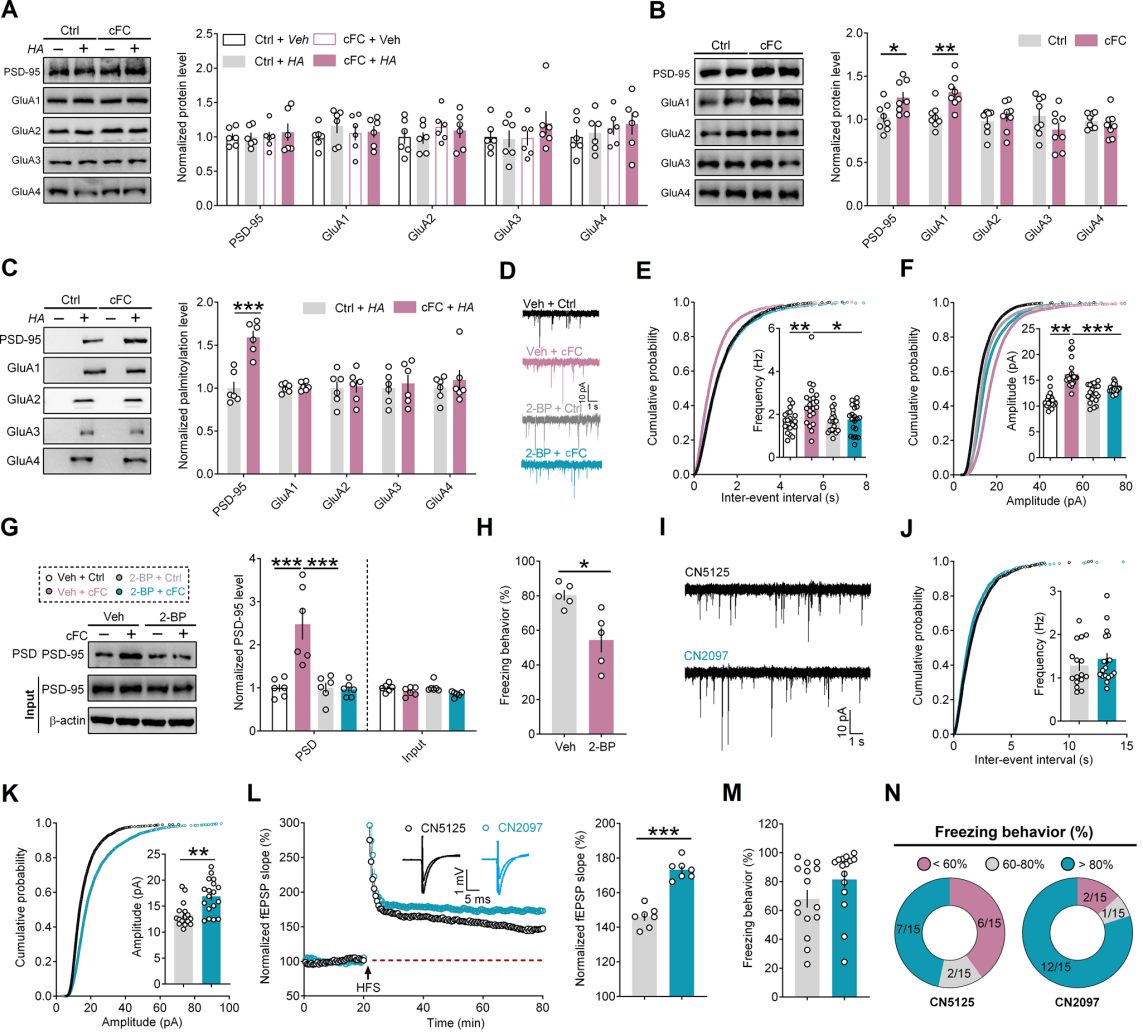
PSD‐95 palmitoylation is required for fear memory formation. (A to C) Representative Western blots (left) and statistical results (right) of total PSD‐95 and GluA1‐4 expression (A), expression in PSD fractions (B), and palmitoylation levels (C) in the LA of rats subjected to cFC (n = 6 rats per group for A and C, n = 8 rats per group for B). (D to F) Representative mEPSCs traces recorded from the LA neurons (D), and cumulative probability distribution and average mEPSCs frequency (E) and amplitude (F) from rats exposed to cFC and/or 2‐BP treatment (n = 20, 21, 19, 20 cells from 6 rats). (G) Representative Western blots (left) and statistical results (right) of total PSD‐95 expression and its enrichment in PSD fraction within the LA of rats exposed to cFC (n = 6 rats per group). (H) Cue fear memory test on Day 3 following 2‐BP microinjection into the LA (n = 5 rats per group). (I–K) Representative mEPSCs traces recorded from the LA neurons of rats treated with CN2097 or CN5125 (I), and cumulative probability and average mEPSCs frequency (J) and amplitude (K) from the CN5125‐ and CN2097‐treated groups (n = 16, 18 cells from 6 rats). (L) Time‐course of HFS‐induced LTP in amygdala slices from the CN5125‐ and CN2097‐treated groups (left), and the histogram showing LTP magnitude averaged from the last 15 min of recordings from different groups (n = 7 slices from 3 rats). (M) Cue fear memory test on Day 3 following CN5125 and CN2097 treatment (n = 15 rats per group). (N) Distribution of rats across different freezing level categories (< 60%, 60–80%, and >80% freezing time (n = 15 rats per group). Data are represented as mean ± SEM. ^*^ *p* < 0.05, ^**^ *p* < 0.01, ^***^ *p* < 0.001. Statistical analyses were performed using one‐way ANOVA followed by Tukey’ s post hoc test (A to C, H to N) and unpaired two‐tailed Student's *t*‐test (E–G).

To further confirm our hypothesis, we first detected the expression of PSD‐95 and AMPARs in the LA after cFC, and found there was no significant change in the expression of PSD‐95 and AMPARs subunits (Figure 3A). In the surface biotinylation PSD purification assay, we observed a significant increase in GluA1 surface expression at the postsynaptic membrane, as well as an increased PSD‐95 expression in the PSDs after cFC (Figure 3B). Next, we performed ABE assay and revealed that the palmitoylation level of PSD‐95 was significantly increased in the LA of cue fear conditioned rats, while little changes in AMPARs palmitoylation were observed (Figure 3C). To examine the role of PSD‐95 palmitoylation in the LA synaptic transmission changes induced by fear conditioning, we recorded the mEPSCs in the presence or absence of 2‐BP (a broad‐spectrum palmitoylation inhibitor) bath application in brain slices from rats exposed to fear conditioning. While acknowledging that 2‐BP has known off‐target effects and inhibits global palmitoylation, our observation that 2‐BP treatment mimicked the molecular phenotype supports the involvement of palmitoylation in this process. We found that cue fear conditioning induced increases in the amplitude and frequency of mEPSCs in the LA, which were abolished by the 2‐BP treatment (Figure 3D‐F). Then, we assessed the impact of 2‐BP on fear induced PSD‐95 expression in the PSDs, and found that 2‐BP abolished the increased PSD‐95 expression in the PSDs, without influence on PSD‐95 expression in the LA after fear conditioning (Figure 3G). Similarly, 2‐BP reversed the GluA1 and GluA3 surface expression at the postsynaptic membrane induced by cue fear conditioning, without influence on GluA2 and GluA4 surface expression (Figure S6). Moreover, we performed cannula implantation and microinjected 2‐BP directly into the LA 30 min before a fear memory test on day 3. We found that the 2‐BP treatment decreased the percentage of freezing time in rats exposed to cue fear conditioning (Figure 3H). These results suggest that fear conditioning leads to increased PSD‐95/AMPARs interaction by hyper‐palmitoylation in the LA that directly impacts LA synaptic transmission and contributes to fear memory expression.

To further test our hypothesis, we utilized a previously reported bridged cyclic peptide, CN2097^40^, to enhance PSD‐95 function at the postsynaptic membrane and performed whole‐cell patch clamp recordings from LA neurons. We observed significant increases in the amplitude of mEPSCs in LA neurons following acute CN2097 bath application, with no significant change in mEPSCs frequency (Figure 3I‐K). Using surface biotinylation assay, we detected increased GluA1 surface expression in the CN2097‐treated group, whereas GluA2‐4 surface expression remained largely unaltered (Figure S7). Given that LTP is an experience‐dependent neural plasticity widely regarded as a cellular substrate for memory formation [41, 42], we recorded the slope of fEPSPs. Results showed that CN2097 significantly increased the LTP induction rate under subthreshold conditions (Figure 3L). These findings support the hypothesis that palmitoylation supports PSD‐95‐mediated enhancement synaptic transmission. To further clarify the role of palmitoylation‐facilitated enhancement of PSD‐95 function at the postsynaptic membrane in fear memory formation, we microinjected either CN5125 (negative control) or CN2097 into the LA region. Intra‐LA infusions of CN2097 did not affect the percentage of freezing time during the fear memory test (Figure 3M). However, on day 3 post‐training, an obvious upward shift was observed in the number of rats with higher fear levels (based on freezing time distribution) in the CN2097 group (Figure 3N). Summary, these results reveal that palmitoylation‐mediated enhancement of postsynaptic PSD‐95 function in the LA is essential for the formation of fear memory.

### 2.4 PSD‐95 Palmitoylation Underlies Learned Fear‐Induced Occlusion of LTP

**FIGURE 4:**
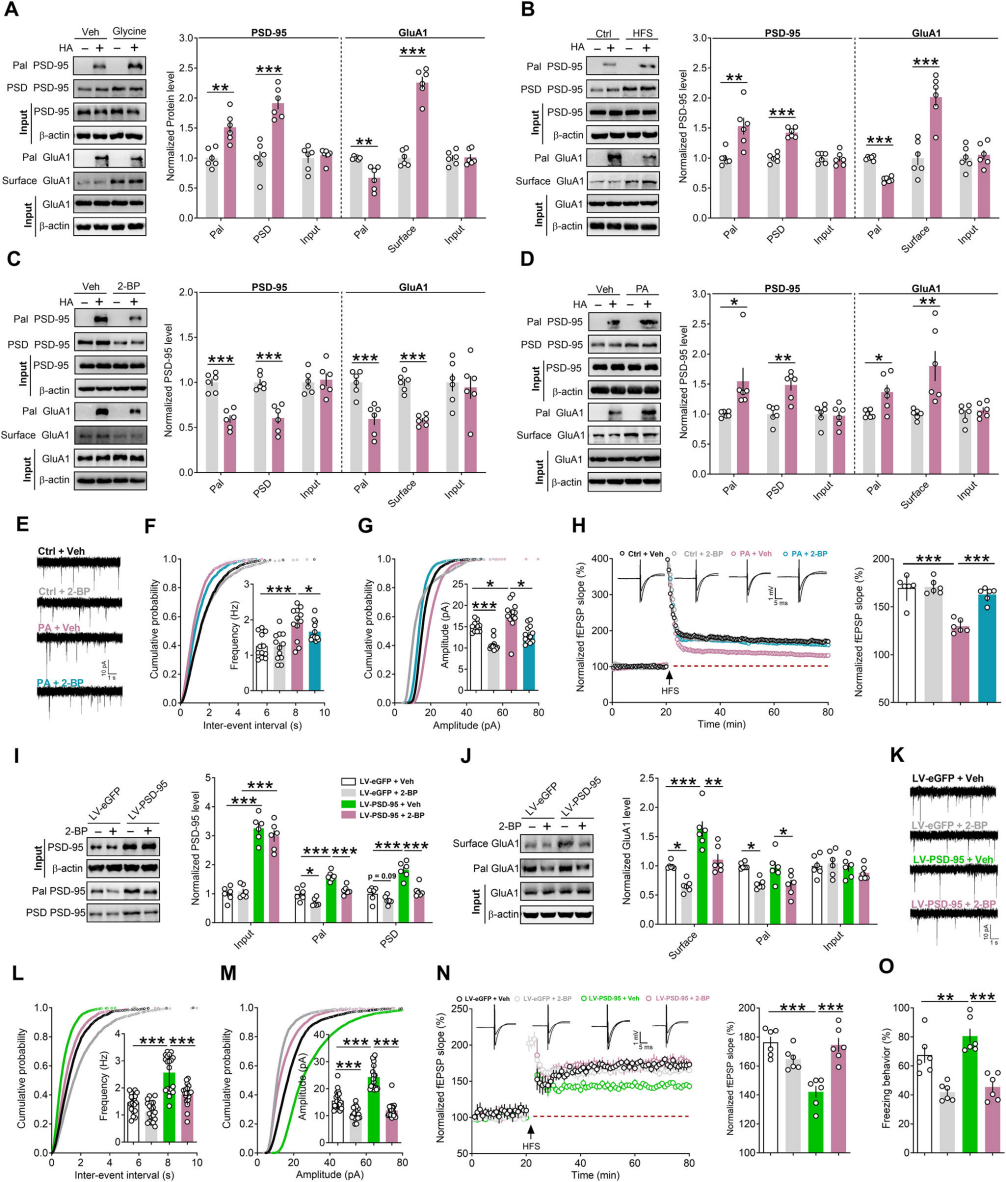
PSD‐95 palmitoylation occludes LA‐LTP following fear conditioning. (A–D) Representative Western blots (left) and quantification (right) of PSD‐95 and GluA1 expression, palmitoylation levels, and postsynaptic membrane surface expression in LA slices subjected to glycine treatment (A), HFS (B), 2‐BP treatment (C), or palmitic acid (PA) treatment (D) (n = 6 rats per group). (E) Representative mEPSCs traces recorded from LA neurons subjected to PA and/or 2‐BP treatment. (F,G) Cumulative probability distributions and average mEPSCs frequency (F) and amplitude (G) from PA and/or 2‐BP treatment groups (n = 12 cells from 6 rats). (H) Time course of HFS‐induced LTP in LA slices from PA and/or 2‐BP treatment groups (left), and histogram showing LTP magnitude averaged from the final 15 min of recordings (right) (n = 6 slices from 3 rats). (I,J) Representative Western blots (left) and quantification (right) of PSD‐95 (I) and GluA1 (J) expression, palmitoylation levels, and postsynaptic membrane surface expression in LA slices transfected with LV‐PSD‐95 and/or treated with 2‐BP (n = 6 rats per group). (K) Representative mEPSCs traces recorded from LA neurons transfected with LV‐PSD‐95 and/or treated with 2‐BP. (L,M) Cumulative probability distributions and average mEPSCs frequency (L) and amplitude (M) from LV‐PSD‐95 and/or 2‐BP treatment groups (n = 20, 19, 19, 20 cells from 8 rats). (N) Time course of HFS‐induced LTP in LA slices from LV‐PSD‐95 and/or 2‐BP treatment groups (left), and histogram showing LTP magnitude averaged from the final 15 min of recordings (right) (n = 6 slices from 3 rats). (O) Cue fear memory test on Day 3 following LV‐PSD‐95 and/or 2‐BP treatment in the LA (n = 6 rats per group). Data are represented as mean ± SEM. ^*^ *p* < 0.05, ^**^ *p* < 0.01, ^***^ *p* < 0.001. Statistical analyses were performed using one‐way ANOVA followed by Tukey’ s post hoc test (A to D) and unpaired two‐tailed Student's *t*‐test (F–O).

Our previous findings, together with recent studies, have shown that learned fear responses occlude electrically induced LTP at LA synapses. Earlier studies also indicate that PSD‐95 mimics and occludes hippocampal LTP while enhancing long‐term depression (LTD) [34, 35]. Thus, we hypothesized that fear learning‐induced hyper‐palmitoylation of PSD‐95 contributes to LTP impairment in the LA. First, we treated amygdala slices with glycine for 5 min to induce the chemical LTP (cLTP), then examined PSD‐95 palmitoylation and its enrichment in the PSD fraction. As shown in Figure 4A, cLTP induction led to significant increases in PSD‐95 palmitoylation and its accumulation in the PSD. Conversely, cLTP reduced GluA1 palmitoylation levels while increasing its surface expression. Consistent results were observed in LTP induced by HFS (Figure 4B). These findings suggest that the enhanced enrichment of PSD‐95 and surface expression of GluA1 at the postsynaptic membrane during LTP are likely attributed to PSD‐95 palmitoylation.

To further confirm the role of PSD‐95 palmitoylation in LTP regulation, we employed 2‐BP to investigate how PSD‐95 palmitoylation affects GluA1 surface expression. As expected, pharmacological blockade of palmitoylation significantly reduced PSD‐95 enrichment in the PSD fraction, along with a concurrent decrease in GluA1 surface expression (Figure 4C). Moreover, to pharmacologically augment global palmitoylation levels, we treated brain slices with palmitic acid (PA). While acknowledging that PA acts as a broad‐spectrum palmitoyl donor that may also alter general lipid metabolism and membrane properties, we observed that PA treatment markedly elevated the palmitoylation levels of both PSD‐95 and GluA1, accompanied by enhanced postsynaptic enrichment of these proteins (Figure 4D), suggesting that palmitoylation facilitates PSD‐95 localization and stability in the PSDs, and thereby it induces the insertion of GluA1 at the postsynaptic membrane. To further dissect the role of PSD‐95 palmitoylation in regulating LA synaptic transmission, we treated LA slices with PA, 2‐BP, followed by recordings of mEPSCs and LTP. We observed a significant increase in both the amplitude and frequency of mEPSCs in PA‐treated slices, an effect that was completely abrogated by co‐treatment with 2‐BP (Figure 4E–G). Notably, we also found that PA treatment led to impaired LTP induction in the LA, a deficit that was rescued by 2‐BP co‐treatment (Figure 4H). Given the broad pharmacological profiles of these agents, these results cannot exclusively isolate the role of PSD‐95. However, they provide crucial functional evidence that a widespread enhancement of palmitoylation statu, mimicking the specific increase in PSD‐95 palmitoylation observed following fear conditioning, is sufficient to drive LTP occlusion in the LA.

Next, we sought to determine whether PSD‐95 overexpression alone is sufficient to recapitulate LTP impairment in the LA. To address this question, we utilized 2‐BP to dissect the impact of palmitoylation on PSD‐95 enrichment in the PSD fraction. We found that 2‐BP completely abolished the AAV‐PSD‐95 mediated increase in PSD‐95 enrichment in the PSD, while exerting minimal effects on the total overexpression level of PSD‐95 (Figure 4I). Similarly, AAV‐PSD‐95 transfection led to a marked elevation in GluA1 surface expression in the LA, and this effect was fully reversed by 2‐BP treatment (Figure 4J). We further evaluated the effects of AAV‐PSD‐95 overexpression on mEPSCs and LTP. Compared with the AAV‐eGFP group, the AAV‐PSD‐95 group exhibited significant increases in both the frequency and amplitude of mEPSCs; notably, this potentiating effect was completely prevented by 2‐BP co‐treatment (Figure 4K–M). Importantly, fEPSP recordings from amygdala slices revealed that application of 2‐BP at the slice level robustly reversed the LTP impairment induced by PSD‐95 overexpression in the LA (Figure 4N). Lastly, we assessed cue fear memory acquisition on day 3, and found that freezing behavior was significantly reduced in rats treated with 2‐BP (1 nmol per side, administered 1 h prior to testing) relative to the control group (Figure 4O). Collectively, these results demonstrate that palmitoylation‐mediated enhancement of PSD‐95 enrichment in the PSD fraction contributes to LTP impairment in the LA.

### 2.5 DHHC2 Mediates Fear Conditioning‐Induced PSD‐95 Palmitoylation

**FIGURE 5:**
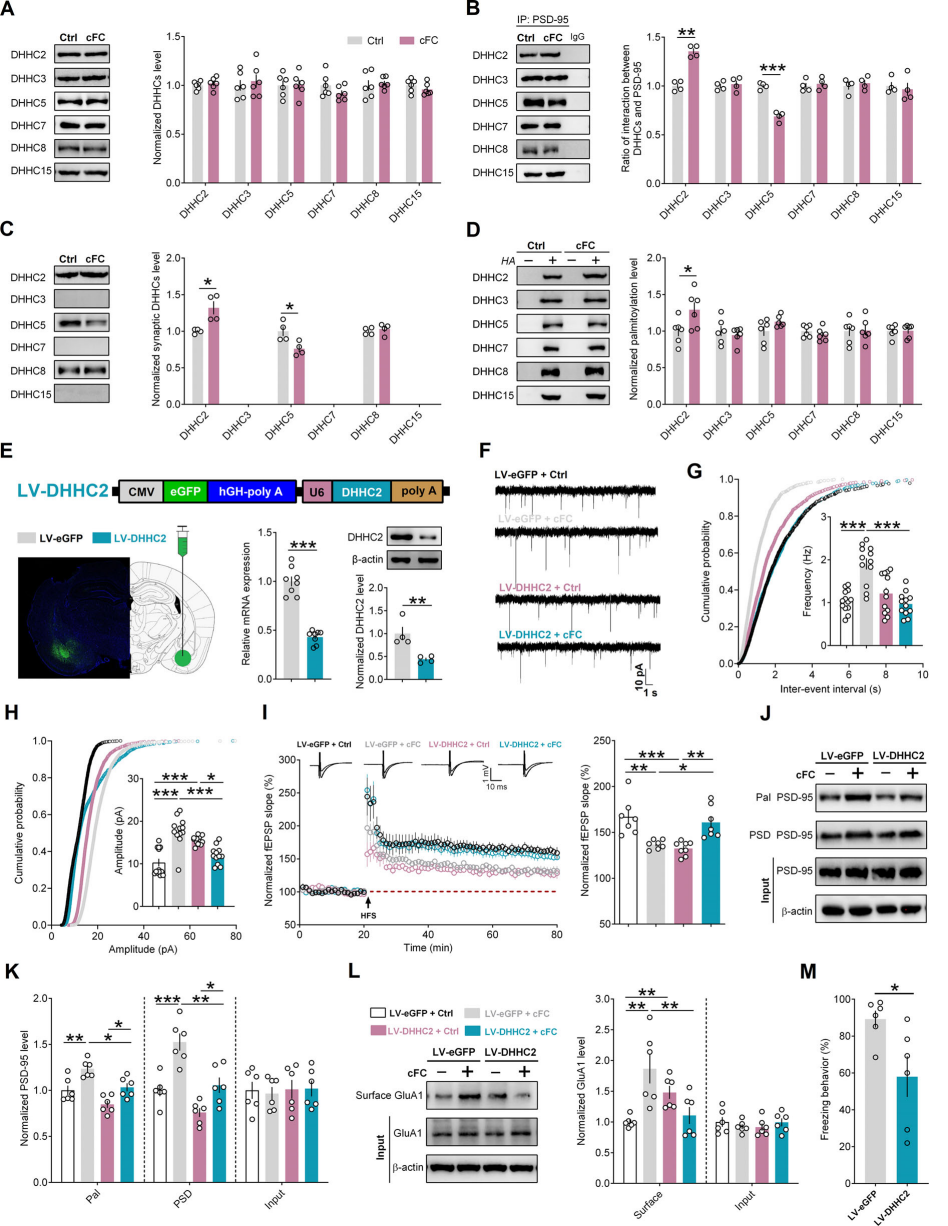
DHHC2 drives PSD‐95 palmitoylation and synaptic plasticity alterations in the LA following fear conditioning. (A to D) Representative Western blots (left) and statistical results (right) of PSD‐95‐associated DHHCs expression (A), co‐immunoprecipitation of PSD‐95 and DHHCs (B), synaptic DHHCs expression (C), and DHHCs auto‐palmitoylation levels (D) in lysates from the LA of rats exposed to cFC. (n = 6 rats per group for A and D, n = 4 rats per group for B and C). (E) Schematic diagram of the LV‐DHHC2 construct, representative stereotaxic injection coordinates, and immunofluorescence staining of coronal brain sections showing the expression of DHHC2 in the LA. Below: Representative Western blots and quantification of DHHC2 protein levels in LA tissues from LV‐eGFP and LV‐DHHC2 groups (n = 4–8 rats per group). (F) Representative mEPSCs traces recorded from LA neurons. (G,H) Cumulative probability distributions and average mEPSCs frequency (G) and amplitude (H) from LV‐DHHC2 and/or cFC groups (n = 13, 12, 12, 12 cells from 6 rats per group). (I) Time course of HFS‐induced LTP in LA slices from LV‐eGFP/LV‐DHHC2‐treated and/or cFC groups (left), with a histogram showing LTP magnitude averaged from the final 15 min of recordings (right) (n = 7–8 slices from 4 rats). (J and K) Representative Western blots (J) and statistical results (K) of PSD‐95 expression, palmitoylation levels and postsynaptic membrane expression in LV‐eGFP/LV‐DHHC2 treated and/or cFC groups. (L) Representative Western blots (left) and statistical results (right) of GluA1 expression and postsynaptic membrane expression in LV‐eGFP/LV‐DHHC2 treated and/or cFC groups (n = 6 rats per group). (M) Cue fear memory test on Day 3 following DHHC2 knockdown in the LA (n = 6 rats per group). Data are represented as mean ± SEM. ^*^ *p* < 0.05, ^**^ *p* < 0.01, ^***^ *p* < 0.001. Statistical analyses were performed using one‐way ANOVA followed by Tukey’ s post hoc test (A to E, and M) and unpaired two‐tailed Student's *t*‐test (G to L).

Our previous findings have demonstrated that PSD‐95 palmitoylation is required for the synaptic clustering of PSD‐95 and the regulation of synaptic strength. However, it remains unclear which member of the DHHC protein family mediates PSD‐95 palmitoylation and thereby contributes to LTP impairment in the LA. Notably, multiple DHHC subfamily members are known to mediate PSD‐95 palmitoylation: DHHC2 and DHHC8 induce PSD‐95 palmitoylation in response to synaptic activity blockade, whereas DHHC3, −7, and −15 drive constitutive PSD‐95 palmitoylation [21]. Intriguingly, DHHC5 is reported to associate with PSD‐95 but lacks PSD‐95 palmitoylation activity [43]. To identify the DHHC isoform responsible for PSD‐95 palmitoylation following fear conditioning, we first performed immunoblotting analysis to assess the expression levels of these DHHC proteins. Unexpectedly, no significant alterations in the expression of these DHHC family members were detected (Figure 5A). We next conducted co‐immunoprecipitation (Co‐IP) assays to examine the interaction between DHHC proteins and PSD‐95. Results showed that fear conditioning markedly enhanced the association between PSD‐95 and DHHC2, while reducing the binding of DHHC5 to PSD‐95 (Figure 5B). To further validate these findings, we purified the PSD fraction via gradient centrifugation and found that LTP induction promoted the trafficking of DHHC2 to PSD‐95‐containing synaptic compartments, accompanied by the dissociation of DHHC5 from the PSD fraction (Figure 5C). These experiments were performed following previously established ABE protocols, which assess DHHC enzyme activity via the detection of auto‐palmitoylation (CSS‐Palm 4.0; http://csspalm.biocuckoo.org) (Figure S8). Using this approach, we found that the auto‐palmitoylation level of DHHC2 was significantly upregulated after cue fear conditioning, whereas no changes in auto‐palmitoylation were observed for DHHC3, −5, −7, −8, or −15 (Figure 5D). These findings demonstrate that DHHC2 plays a critical role in mediating PSD‐95 palmitoylation during fear conditioning.

To further confirm the role of DHHC2 in the regulation of synaptic plasticity mediated by fear learning, we first constructed a lentiviral short hairpin RNA targeting DHHC2 for delivery to the LA, aiming to achieve DHHC2 knockdown. Quantitative validation via RT‐qPCR and immunoblotting demonstrated that DHHC2 mRNA and protein levels were significantly reduced in the LA of LV‐shRNA‐DHHC2‐injected rats relative to LV‐eGFP controls, confirming efficient DHHC2 knockdown (Figure 5E). To assess how DHHC2 knockdown modulates basal synaptic transmission, we recorded mEPSCs in LA neurons, and found that fear conditioning elevated both mEPSCs amplitude and frequency in the LV‐eGFP group. Notably, DHHC2 knockdown reversed these cFC‐driven changes: mEPSCs amplitude and frequency were restored to near‐baseline levels (Figure 5F–H). We next assessed synaptic plasticity by quantifying normalized fEPSP slopes following HFS. Consistent with our previous observations, fear conditioning induced a significant reduction in fEPSP slopes in the LV‐eGFP group relative to the unconditioned control group. DHHC2 knockdown via shRNA robustly reversed this fear conditioning‐induced LTP impairment (Figure 5I). Combining ABE assay with PSD fraction purification, we found that the fear conditioning‐induced elevation in PSD‐95 palmitoylation was abrogated by DHHC2 silencing in the LA; a corresponding reversal was also detected for fear conditioning‐driven PSD‐95 enrichment at the postsynaptic membrane (Figure 5J,K). Subsequently, we quantified surface expression of AMPARs subunit GluA1 using a surface biotinylation assay. In the LV‐eGFP group, cFC increased surface GluA1 levels compared to controls; however, DHHC2 knockdown reversed this cFC‐induced upregulation—consistent with the restored mEPSCs dynamics noted earlier (Figure 5L). Lastly, following microinjection of LV‐shRNA‐DHHC2 into the LA region, the percentage of freezing behavior was markedly reduced (Figure 5M). These findings demonstrate that DHHC2‐mediated PSD‐95 palmitoylation is a key driver of LTP impairment in the LA following fear conditioning.

ABHD17 is a known depalmitoylating enzyme that selectively reduces the palmitoylation and synaptic clustering of PSD‐95 [44]. We thus investigated its potential role in fear conditioning. First, we assessed ABHD17 expression via RT‐qPCR and found no significant alterations in the expression of its three subunits in the LA of cue fear‐conditioned rats (Figure S9A). Next, to explore whether palmitate cycling of PSD‐95 contributes to synaptic strength regulation, we overexpressed ABHD17B in the LA via adeno‐associated virus (AAV) delivery, and validated its overexpression using RT‐qPCR and immunoblotting (Figure S9B–D). We then evaluated the impact of ABHD17B overexpression on mEPSCs: ABHD17B upregulation reduced mEPSCs amplitude, with no significant effect on mEPSCs frequency (Figure S9E–G). Consistent with the effects of DHHC2 knockdown in the LA, ABHD17B overexpression did not alter the enhanced total PSD‐95 expression induced by cue fear conditioning, but abrogated both the conditioning‐driven increase in PSD‐95 palmitoylation and the elevated postsynaptic membrane enrichment of PSD‐95 (Figure S9H,I). Finally, following microinjection of AAV‐ABHD17B into the LA region, the percentage of freezing behavior was markedly reduced (Figure S9J). Collectively, these findings further demonstrate that DHHC2‐mediated PSD‐95 palmitoylation cycling contributes to fear conditioning‐induced LTP impairment in the LA.

### 2.6 PICK1 Regulates the Activity‐Dependent Trafficking of DHHC2 Toward PSD‐95 at Synapses

**FIGURE 6:**
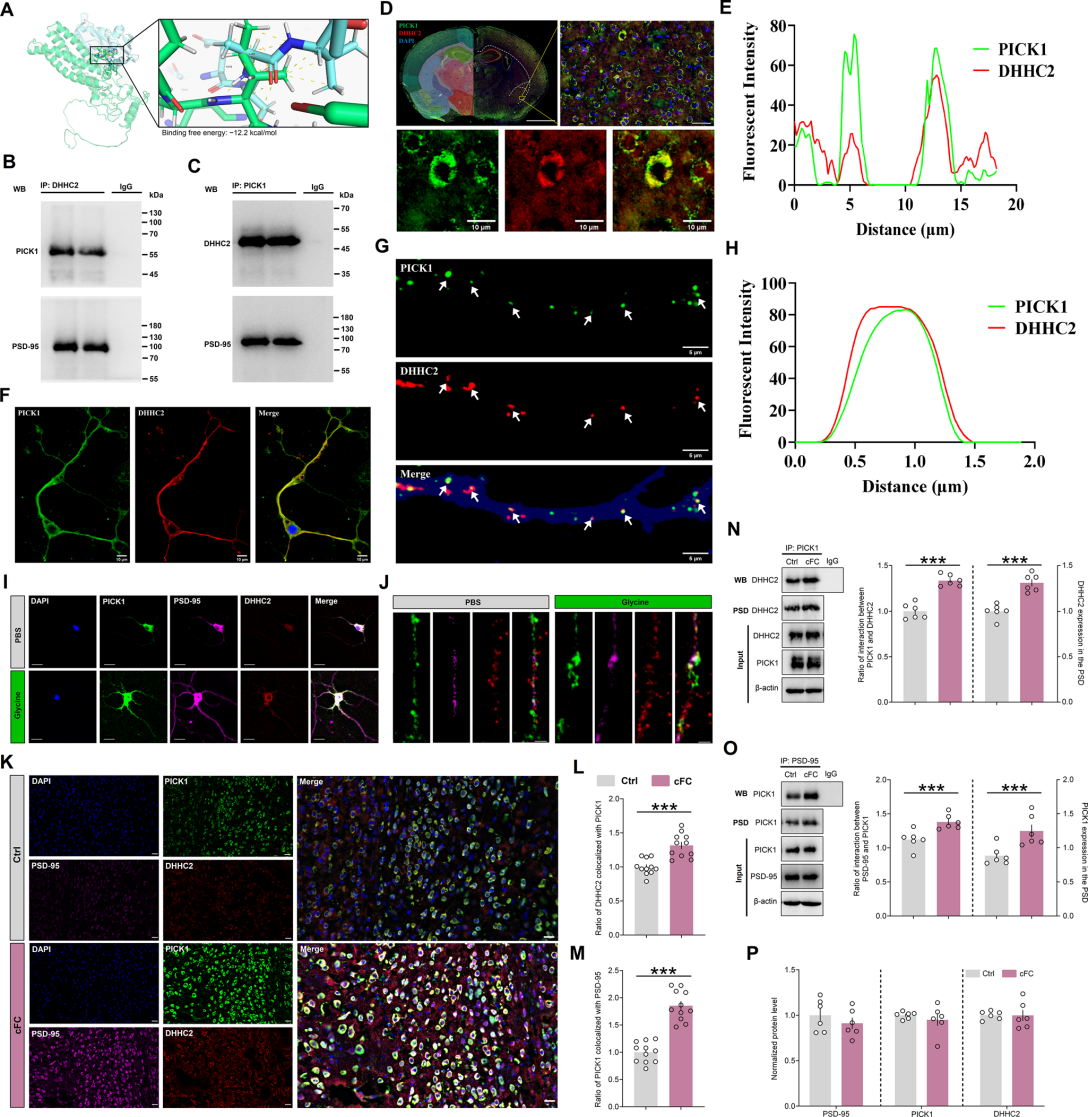
PICK1 mediates activity‐dependent trafficking of DHHC2 to PSD‐95. (A) Predicted binding affinity of the PICK1‐DHHC2 complex, showing a strong physical interaction (ΔG = −12.2 kcal·mol^−1^; K_d ≈ 1.2 × 10^−9 ^M), predominantly driven by hydrophobic interfacial interactions. (B,C) Representative Western blots from co‐immunoprecipitation (Co‐IP) assays, confirming the physical interactions among PICK1, DHHC2, and PSD‐95 in the LA. (D,E) Representative immunofluorescence staining (D) and fluorescent intensity analysis (E) showing co‐localization of PICK1 and DHHC2 in the rat LA: Top left, immunofluorescence image of brain coronal section highlighting the LA region; Top right, high‐magnification view of the LA; Bottom panels, channel‐separated (PICK1: green; DHHC2: red) and merged views of a single LA neuron (yellow indicates co‐localization; DAPI: blue, nuclear stain). Scale bars: 10 µm. (F–H) Representative immunofluorescence staining of PICK1 and DHHC2 subcellular co‐localization in cultured hippocampal neurons (F; Scale bar: 10 µm) and high‐magnification views revealing punctate co‐localization of PICK1 and DHHC2 in neuronal processes (G; Scale bar: 10 µm). (I,J) Representative immunofluorescence staining showing glycine treatment modulates the subcellular localization and co‐localization of PICK1, DHHC2, and PSD‐95 in cultured hippocampal neurons (PBS as control; Scale bar: 10 µm). (K) Representative immunofluorescence staining of PICK1 co‐localization with DHHC2 and PSD‐95 in LA tissue sections from Ctrl and cFC‐treated rats (Scale bar: 50 µm). (L,M) Quantitative analysis of the co‐localization ratio of DHHC2 with PICK1 (L) and PICK1 with PSD‐95 (M) in Ctrl vs. cFC groups (n = 11 rats per group). (N) Representative Co‐IP and Western blot results (left) alongside quantitative analysis (right): These show the interaction between PICK1 and DHHC2 (detected via PICK1‐targeted Co‐IP), DHHC2 expression in PSD‐enriched fractions, and total PICK1/DHHC2 expression in LA lysates, from Ctrl and cFC‐treated rats (IgG as negative control; n = 6 rats per group). (O) Representative Co‐IP and Western blot results (left) alongside quantitative analysis (right): These show the interaction between PSD‐95 and PICK1 (detected via PSD‐95‐targeted Co‐IP), PICK1 expression in PSD‐enriched fractions, and total PSD‐95/PICK1 expression in LA lysates, from Ctrl and cFC‐treated rats (IgG as negative control; n = 6 rats per group). (P) Quantitative analysis of PICK1, DHHC2, and PSD‐95 expression levels in LA lysates (n = 6 rats per group). Data are represented as mean ± SEM. ^**^ *p* < 0.01, ^***^ *p* < 0.001. Statistical analyses were performed using one‐way ANOVA followed by Tukey’ s post hoc test.

Our preceding findings demonstrate that DHHC2 localizes to dendrites and somata in the form of small vesicular puncta, translocates to the PSD in response to synaptic activity blockade [45], and mediates activity‐dependent PSD‐95 palmitoylation [46]. However, the molecular mechanism underlying the trafficking of dendritic DHHC2 to the PSD remains to be elucidated. Our aforementioned data reveal that the molecular mechanism mediating DHHC2 trafficking must satisfy two core criteria: first, it must be capable of rapidly sensing and responding to dynamic changes in neuronal activity; second, it must be able to directly bind to DHHC2 and act as a carrier to achieve its targeted delivery to the postsynaptic compartment. It can thus be reasonably inferred that protein interacting with C‐kinase 1(PICK1) represents the most promising candidate molecule mediating this trafficking process. To verify this hypothesis, we first used molecular docking to predict the interaction between PICK1 and DHHC2, which revealed a stable binding mode with a binding energy of −12.2 kcal/mol (Figure 6A), indicating a high‐affinity interaction between the two proteins. Co‐IP assays further confirmed that DHHC2, PICK1, and PSD‐95 form a ternary complex in vivo: immunoprecipitation of DHHC2 pulled down both PICK1 and PSD‐95 (Figure 6B), and immunoprecipitation of PICK1 similarly pulled down DHHC2 and PSD‐95 (Figure 6C). Furthermore, confocal immunofluorescence imaging showed that PICK1 and DHHC2 co‐localized in the cytoplasm of transfected cells (Figure 6D), with fluorescence intensity profiles confirming overlapping signal peaks (Figure 6E). In primary neurons, PICK1 and DHHC2 co‐localized along neuronal processes (Figure 6F), and high‐magnification imaging revealed their co‐localization at PSD‐95‐positive synaptic sites (Figure 6G). Fluorescence intensity line scans across individual synapses showed synchronized peaks of PICK1 and DHHC2 signals, indicating their precise co‐localization at synaptic compartments (Figure 6H). These results suggest that PICK1 and DHHC2 co‐localize at synaptic compartments.

**FIGURE 7:**
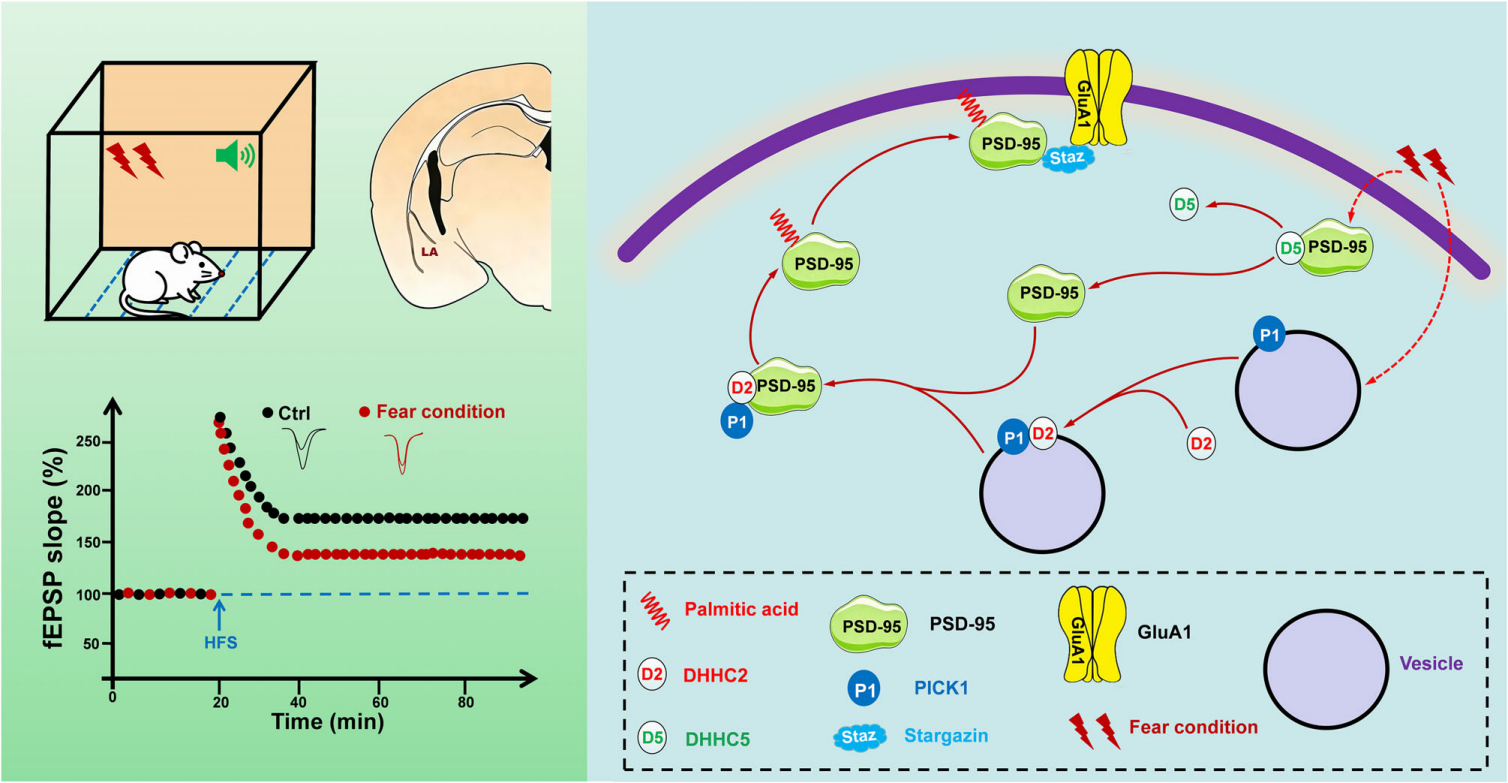
A schematic diagram of PICK1‐DHHC2‐PSD‐95 palmitoylation signaling involved in fear conditioning. Fear stress‐induced neuronal activation disrupts the endogenous interaction between DHHC5 and PSD‐95 at PSD and triggers DHHC5 endocytosis. Concurrently, neuronal activity drives PICK1, acting via its molecular adaptor function, to mediate the targeted trafficking of DHHC2 toward PSD‐95, thereby triggering PSD‐95 palmitoylation and its subsequent trafficking to dendritic spines. Within spines, PSD‐95 palmitoylation not only enhances its own synaptic stabilization but also selectively facilitates the surface recruitment and retention of GluA1‐subtype AMPA receptors at postsynaptic membrane. Collectively, these events induce a state of learning‐dependent metaplasticity in lateral amygdala circuits, which occludes further HFS‐induced LTP while promoting fear memory formation.

To investigate how neuronal activity modulates the interaction and synaptic localization of the PICK1‐DHHC2‐PSD‐95 complex, we first treated cultured hippocampal neurons with glycine to enhance synaptic activity, and subsequently performed triple immunofluorescence staining, Co‐IP assay, and PSD fractionation assay. As expected, glycine stimulation induced a rapid increase in PICK1 binding to both DHHC2 and PSD‐95 (Figure 6I,J). Consistent with this, Co‐IP assays using an anti‐PICK1 antibody revealed that glycine treatment significantly increased the amount of DHHC2 co‐immunoprecipitated with PICK1 relative to PBS controls; similar effects were observed in Co‐IP assays using an anti‐PSD‐95 antibody (Figure S10). These in vitro results demonstrate that the activity‐driven formation of the PICK1‐DHHC2‐PSD‐95 complex is a fundamental molecular capacity present in neurons. However, whether this conserved molecular capacity is differentially engaged by distinct physiological stimuli in specific brain regions in vivo remains unclear. To assess whether fear conditioning modulates the assembly of this complex in vivo, we performed immunofluorescence staining in the LA of control and fear conditioning‐treated rats. cFC significantly increased the co‐localization of DHHC2 with PICK1 and of PICK1 with PSD‐95 in the LA, as quantified by Manders’ overlap coefficient (Figure 6K–M). Co‐IP assays further confirmed that cFC enhanced the interaction between DHHC2 and PICK1, as well as between PICK1 and PSD‐95, in the LA (Figure 6N,O). Notably, total protein levels of PSD‐95, PICK1, and DHHC2 in whole‐cell lysates remained unchanged across groups (Figure 6P). Collectively, these findings demonstrate that fear conditioning drives the synaptic assembly of the PICK1‐DHHC2‐PSD‐95 complex, linking in vitro neuronal activity to in vivo fear‐related synaptic plasticity (Figure 7).

## 3 Discussion

Protein palmitoylation is implicated in learning and memory, but the precise mechanisms governing its contribution to fear memory formation remain unclear. Employing a multifaceted approach combining electrophysiological recordings, molecular biological techniques, and behavioral cue fear conditioning assays, we tested the hypothesis that fear conditioning induces PSD‐95 palmitoylation through PICK1‐dependent DHHC2 translocation—selectively stabilizing GluA1 at amygdalar synapses and constraining LTP. We uncovered a region‐specific modulation of synaptic plasticity: hippocampal LTP remains unaltered, while LA LTP is significantly impaired. This dissociation is consistent with classical fear memory models, indicating that amygdalar circuits attain learning‐dependent saturation (restricting further LTP induction) whereas hippocampal circuits retain synaptic potentiation capacity—refining existing frameworks of fear learning. We further verified that palmitoylation is essential for synaptic transmission at the network level and delineated its role in regulating LA LTP impairment following fear memory acquisition, mediated by crosstalk with synaptic plasticity. We propose that this dissociation occurs because amygdalar circuits, driven by excessive PSD‐95 accumulation, enter a state of synaptic structural saturation that restricts further LTP induction (metaplastic impairment), whereas hippocampal circuits retain their synaptic remodeling capacity—refining existing frameworks of fear learning. Collectively, our findings define a novel mechanism whereby aberrant palmitoylation induces LA LTP occlusion and synaptic plasticity impairment, and underscore the PICK1‐DHHC2‐PSD‐95 pathway as a promising therapeutic target for PTSD and other fear‐related anxiety disorders.

Numerous studies have demonstrated that palmitoylation regulates the stability, sorting, and trafficking of neuronal proteins, thereby modulating their synaptic targeting and functional efficacy [20, 47]. In recent years, a growing body of evidence has revealed that synaptic protein palmitoylation is activity‐dependent, involving key molecules such as PSD‐95 [48], AMPA receptors (AMPARs) [49], and δ‐catenin [50]. Our works showed that fear conditioning‐induced hyperactivity in the LA was accompanied by increased global palmitoylation; notably, blocking neuronal activity with TTX (to mimic enhanced synaptic network strength) further augmented global palmitoylation—including that of PSD‐95, and consistent findings have been observed in hippocampal neurons following fear conditioning [51]. Additionally, an earlier study demonstrated that acute increases in neuronal activity enhance AKAP150 palmitoylation, stabilizing it in the membrane via association with lipid rafts [52, 53]. Homeostatic elevations in neuronal activity have also been shown to enhance PSD‐95 palmitoylation for up to 180 min [50]. Together, these observations suggest that palmitoylation of most synaptic proteins contributes to synaptic plasticity, leading to relatively stable modifications of synaptic molecules. However, the causal role of synaptic protein palmitoylation in synaptic plasticity remains unexplored. Here, we used 2‐BP to validate palmitoylation's role in fear conditioning‐induced changes in LA synaptic transmission, finding that 2‐BP abolished the fear conditioning‐mediated mEPSCs amplitude and frequency. This aligns with our recent work showing that treatment with palmitoylation inhibitors (2‐BP or NtBuHA) reverses the fear conditioning‐induced upregulation of palmitoylation in target molecules (PSD‐95, GluA1‐2, and NMDARs) and reduces the slope of hippocampal LTP [51]. Collectively, these data indicate clear bidirectional crosstalk between protein palmitoylation and synaptic plasticity.

PSD‐95 is the most extensively studied scaffolding protein in the context of synaptic plasticity and has been implicated in maintaining and regulating glutamatergic synaptic function and dendritic spine morphology [54, 55, 56]. Several studies have reported enhanced LTP in PSD‐95‐deficient rats, and PSD‐95 itself can mimic and occlude LTP in the cerebral cortex and hippocampus [57, 58, 59]. In contrast, a growing body of research has demonstrated that PSD‐95 overexpression increases the number of postsynaptic AMPARs, facilitating LTP expression [33, 54, 60]. While these findings remain controversial, these relevant correlative observations indicate that PSD‐95 dynamics are closely associated with LTP expression. Notably, blocking APT1 (a depalmitoylating enzyme) was shown to enhance PSD‐95 palmitoylation, which in turn upregulates both the number of AMPARs binding slots and PSD‐95 levels in the postsynaptic density [51]. Nevertheless, direct evidence that alterations in PSD‐95 palmitoylation status mediate LTP‐related synaptic changes is lacking. Here, we show that global palmitoylation and PSD‐95‐specific palmitoylation levels are significantly elevated following fear‐induced LTP, with no changes in the expression levels of cognate palmitoyltransferases (PATs) or depalmitoylating enzymes.

According to previous reports, DHHC2 and DHHC3 are the major PATs for PSD‐95, and their PAT activity—reflected by auto‐palmitoylation—is regulated by synaptic activity [61, 62]. We therefore examined PAT activity following LTP using the ABE assay and found that the auto‐palmitoylation levels of DHHC2 and DHHC3 remained unaltered. Notably, DHHC3 is specifically localized to the somatic Golgi apparatus [63], whereas DHHC2 resides in mobile vesicles within dendrites and the cell body [64]. Consistent with this subcellular distribution, our findings demonstrate that fear conditioning promotes the dissociation of DHHC5 from PSD‐95 and the subsequent association of PSD‐95 with DHHC2. Furthermore, we found that DHHC2 knockdown significantly decreased the slope of fEPSPs in the LA, and silencing DHHC2 in the LA impaired LTP‐induced translocation of PSD‐95 to the postsynaptic density. In line with these observations, overexpression of ABHD17B in the LA selectively reduced PSD‐95 palmitoylation, as well as the synaptic clustering of both PSD‐95 and AMPARs. Collectively, our findings demonstrate that DHHC2‐mediated palmitoylation of PSD‐95 is essential for the expression of LA‐LTP.

Previous studies on LTP have demonstrated that fear conditioning is associated with LTP‐like modifications in thalamo‐amygdala synaptic transmission—a pathway that conveys specific conditioned stimulus information to the LA during discriminative fear learning [65, 66]. Notably, recent findings reveal that fear conditioning occludes HFS‐induced LTP in the cortico‐LA pathway [29]. However, the molecular mechanisms underlying the phenotypic convergence between fear conditioning and LTP remain elusive. Prior work has shown that PSD‐95 overexpression profoundly modulates synaptic function, increasing the amplitude of mEPSCs by up to fourfold [67]; importantly, synaptic potentiation induced by PSD‐95 overexpression cannot be further enhanced by HFS protocols [35]. Elevated PSD‐95 expression mimics and occludes hippocampal LTP while enhancing LTD [34]. Mechanistically, reduced PSD‐95 palmitoylation mediates the regulatory effect of Ca^2^^+^/CaMKII on PSD‐95 synaptic localization during LTD [68]. Our recent work further demonstrated a marked increase in PSD‐95 palmitoylation during cLTP, an effect abrogated by treatment with the palmitoylation inhibitor 2‐BP [51]. Collectively, these observations suggest that palmitoylation regulates the dynamic recruitment of PSD‐95 to the postsynaptic membrane and modulates synaptic transmission. In contrast, excessive PSD‐95 accumulation at the postsynaptic density occludes LTP induction. To test this hypothesis, we examined the interaction between PSD‐95 and DHHC palmitoyltransferases. We found that fear conditioning induces a significant increase in both DHHC2 and PSD‐95 palmitoylation. Notably, DHHC2 silencing rescued the LTP impairment induced by fear conditioning, whereas basal LTP was impaired in DHHC2 knockdown rats. Together, our results demonstrate that PSD‐95 palmitoylation is critical for synaptic transmission and that palmitoylation‐dependent accumulation of excessive PSD‐95 at the postsynaptic membrane underlies LTP occlusion in the LA following fear conditioning. Notably, our observation of LTP occlusion at 2 h post‐fear conditioning temporally overlaps with the well‐characterized, transient insertion of calcium‐permeable AMPA receptors (CP‐AMPARs) in the LA [69]. However, we propose that these represent parallel, mechanistically distinct processes. Functionally, CP‐AMPAR insertion defines a “labilized” state primed for further plasticity, which is conceptually opposite to the “saturated”, non‐plastic state induced by PSD‐95 hyper‐palmitoylation observed here. Furthermore, at the cell biological level, while palmitoylation of AMPAR subunits themselves drives their retrograde trafficking to the Golgi apparatus and reduces surface expression [39], synaptic enrichment of GluA1 in our model is driven by the palmitoylation of the PSD‐95 scaffold, which anchors and stabilizes AMPARs at the PSD. Consistent with the predominance of GluA2‐containing, calcium‐impermeable AMPARs (CI‐AMPARs) at mature synapses, this PSD‐95‐dependent anchoring preferentially stabilizes CI‐AMPARs at existing postsynaptic sites. Therefore, rather than mediating CP‐AMPAR dynamics, the PICK1/DHHC2/PSD‐95 axis likely acts on a separate receptor pool to structurally lock a subset of synapses into a saturated state during early fear memory consolidation.

PICK1, a key molecule in the regulation of synaptic function [70], has been partially confirmed to play roles in AMPARs trafficking, synaptic plasticity, and learning and memory [71, 72]. However, the present study is the first to reveal a novel mechanism by which PICK1 specifically mediates amygdala synaptic plasticity associated with fear memory through regulating the palmitoylation modification pathway. Core findings demonstrate that under fear conditioning induction, PICK1 mediates the targeted trafficking of DHHC2 to PSD‐95 in a manner dependent on its molecular adaptor function, thereby triggering PSD‐95 palmitoylation—this process is not a generalized synaptic modification but selectively stabilizes the GluA1 subtype of AMPARs at the postsynaptic membrane of the amygdala, providing a precise molecular regulatory paradigm for fear memory‐related synaptic plasticity. Integrating our biochemical and behavioral data, we propose that the PICK1‐DHHC2‐PSD‐95 signaling axis functions as a dynamic molecular switch governing metaplasticity across different phases of fear memory: i) During fear conditioning (Synaptic saturation phase), PICK1‐mediated DHHC2 recruitment drives hyper‐palmitoylation, locking PSD‐95/GluA1 into the synapse. This pushes the LA synapse into a state of functional saturation, limiting subsequent LTP induction. ii) During successful extinction (Metaplastic reset phase), we hypothesize that a reconfiguration of this ternary complex occurs. This would facilitate PSD‐95 turnover, relieve the structural saturation, and reset metaplasticity to allow LTP restoration. iii) Crucially, in extinction‐deficit animals (Pathological rigidity phase), this regulatory shift appears to fail. Persistent PICK1‐DHHC2 coupling results in irreversible PSD‐95 retention, maintaining the synapse in a rigid, saturated state. This failure to structurally reset metaplasticity provides a plausible explanation for the persistent LTP impairment and intractable fear memory observed in these individuals.

Extending this molecular model to a structural level, it is plausible that the mechanistic divergence between Ext and Ext deficit groups lies in distinct dendritic spine dynamics. PSD‐95 is a core scaffold of mature, mushroom‐type spines, and its enrichment is closely associated with spine stability and longevity *in* vivo [56]. We therefore infer that persistent, palmitoylated PSD‐95 retention in Ext deficit animals may sustain an overabundance of morphologically rigid, “memory” spines, consistent with reports that stress‐induced extinction deficits are accompanied by increased mushroom spine density and dendritic retraction in basolateral amygdala neurons [73]. In contrast, successful extinction might involve a structural transition–from stabilized, large‐head spines toward more slender, plastic morphologies–resembling cue‐specific elimination of conditioning‐induced new spines observed in the auditory cortex following fear extinction [74]. While our current data do not include direct spine imaging, this structural saturation framework provides a testable model linking PSD‐95 dynamics, metaplastic impairment, and extinction deficits.

In conjunction with previous research, PICK1 has been shown to regulate AMPARs endocytosis and synaptic remodeling through direct binding to GluA2/3 [75]. The present study extends its functional dimension—namely, PICK1 can target the GluA1 subtype and stabilize synaptic function by indirectly regulating the palmitoylation pathway. This suggests that PICK1 may flexibly regulate synaptic plasticity under different physiological and pathological conditions through a dual mode of “direct binding” and “indirect regulation.” Furthermore, this mechanism provides a new perspective for understanding “synaptic function consolidation” (e.g., LTP occlusion) during fear memory formation: PICK1‐mediated GluA1 stabilization may drive amygdalar synapses into a state of functional saturation, limiting subsequent LTP induction, which is highly consistent with the region‐specific differences in synaptic plasticity following fear memory formation (impaired LTP in the amygdala versus preserved LTP in the hippocampus).

Several limitations should be noted. First, we did not explicitly measure shock sensitivity or auditory perception following intra‐LA 2‐BP infusion, though this concentration does not impair general locomotion. Second, a significant limitation of our study is the lack of genetic validation using palmitoylation‐deficient PSD‐95 mutants. While our data strongly support the involvement of the PICK1‐DHHC2 pathway, pharmacological inhibition and enzyme knockdown cannot definitively exclude the contribution of other DHHC2 substrates to establish absolute substrate‐specific causality. Proving this definitively in vivo requires an inducible, conditional PSD‐95 C3S/C5S knock‐in mouse model; a global knock‐in is unfeasible due to the critical role of PSD‐95 palmitoylation in early developmental synaptic organization [76, 77], which would confound the interpretation of adult fear memory phenotypes. Similarly, simple in vitro mutant overexpression cannot recapitulate the complex, activity‐dependent in vivo fear conditioning process. The generation and characterization of the conditional knock‐in model is currently underway in our laboratory and will be the focus of a future study to definitively isolate catalytic from scaffolding functions and establish the causal role of PSD‐95 palmitoylation in fear memory. Third, while activity‐driven complex assembly reflects a conserved molecular capacity in cultured hippocampal neurons, this does not translate to “in vivo” hippocampal metaplasticity. Because hippocampal LTP remained unaffected by manipulations that robustly induced metaplasticity in the LA (Figure 1), mere complex formation is insufficient; LA‐specific microenvironments or afferent inputs likely dictate regional susceptibility. Finally, our use of exclusively male rats—while avoiding estrous cycle‐induced variability in baseline plasticity and molecular interactions—limits generalizability. Given well‐documented sex differences in fear memory and lipid modifications, determining whether this palmitoylation module operates identically in females remains a critical step for clinical translation.

In conclusion, our study establishes a critical role of DHHC2‐mediated PSD‐95 palmitoylation in dynamically regulating synaptic glutamate receptors and synaptic plasticity, and provides a novel mechanistic explanation for LTP impairment in the LA of fear‐conditioned rats. This finding is further supported by the observation of significant upregulation of the interaction between DHHC2 and PICK1 following fear conditioning. Collectively, these results strongly demonstrate that the PICK1/DHHC2/PSD‐95 palmitoylation signaling pathway—which mediates the membrane surface stability of AMPARs—plays a pivotal role in fear memory formation. Importantly, this pathway may represent a promising therapeutic target for the prevention and treatment of PTSD and other fear‐ and anxiety‐related memory disorders.

## 4 Materials and Methods

### 4.1 Animals

Male Sprague Dawley (SD) rats (7–8 weeks old, 200–250 g) were obtained from the Shanghai Laboratory Animal Center, Chinese Academy of Sciences (Shanghai, China). Pregnant female Sprague Dawley (SD) rats (gestational day 18–19, 250–300 g) were obtained from Fujian Medical University. All animals were individually housed in standard cages under a controlled 12‐h light/12‐h dark cycle, with a constant temperature (22 ± 2°C) and relative humidity (55%–65%). Standard laboratory chow and water were provided ad libitum. The care and use of experimental animals in this study complied with the Guide for the Care and Use of Laboratory Animals (National Institutes of Health, NIH, USA). All experimental protocols were approved by the Animal Welfare Committee of Fujian Medical University.

### 4.2 Agents

Tetrodotoxin (TTX), 2‐bromopalmitic acid (2‐BP), hydroxylamine (HA) hydrochloride, S‐methyl methanethiosulfonate (MMTS), neocuproine, and streptavidin‐agarose were purchased from Sigma–Aldrich (St. Louis, MO, USA). N‐(6‐(Biotinamido)hexyl)‐3’‐(2’‐pyridyldithio) propionamide (HPDP‐biotin) was purchased from Thermo Fisher Scientific (Waltham, MA, USA). Synthetic peptides (CN5125 and CN2097) were obtained from Jier (Shanghai, China). All other reagents and kits were purchased from commercial sources.

### 4.3 Cue Fear Conditioning (cFC)

The cFC procedure has been described in our previous study with slight modifications [78]. The cue fear conditioning experiment was performed over 3 consecutive days: Day 1 (habituation), Day 2 (fear conditioning), and Day 3 (testing). On Day 1, rats were individually exposed to the conditioning chamber for 3 min. On Day 2, following 3 min of habituation in the chamber (Context A), rats received three presentations of a tone conditioned stimulus (CS; 80 dB, 29 s), each co‐terminated with a 0.75‐mA foot shock (1 s duration) as the unconditioned stimulus (US), with an intertrial interval of 30 s between consecutive CS–US pairings. Rats were immediately removed from the chambers after the conditioning session. On Day 3, rats were placed in a distinct chamber (Context B) and exposed to five CS presentations (80 dB, 29 s) over a 3‐min test session. Freezing time percentage was quantified using ANY‐maze software (Stoelting Co., Wood Dale, IL, USA). As described in previous studies [30, 79], rats displaying a pronounced reduction in freezing relative to initial extinction training were assigned to the extinction (Ext) group. Conversely, rats whose average freezing duration remained above the median (50%) were categorized into the extinction deficit (Ext deficit) group.

### 4.4 Stereotaxic Injections

Rats were anesthetized with isoflurane (Shenzhen RWD Life Science) and placed in an automated stereotaxic instrument (RWD68001, Shenzhen RWD). Stainless steel guide cannulas (15 mm length; 0.6 mm outer diameter [OD]) were bilaterally implanted into the LA at the following coordinates: anteroposterior (AP) −2.2 mm, mediolateral (ML) ± 5.0 mm, dorsoventral (DV) −8.5 mm (relative to bregma). Cannulas were affixed to the skull using stainless steel jeweler's screws and dental acrylic. Rats were individually housed and allowed a recovery period of at least 7 days post‐surgery.

PSD‐95‐binding peptides (CN5125 and CN2097) were dissolved in dimethyl sulfoxide (DMSO) and further diluted in artificial cerebrospinal fluid (aCSF) to a final concentration of 2 µm. The general protein palmitoylation inhibitor 2‐BP was dissolved in DMSO and diluted in aCSF to a final concentration of 0.6 mm. These reagents were bilaterally infused into the LA via the implanted cannulas at a rate of 0.5 µL/min (total volume: 0.5 µL per side) using a 5‐µL microsyringe connected to a 16‐mm, 0.5‐mm OD injection needle via 10‐cm polyethylene (PE‐10) tubing. Infusion volume and rate were controlled by an injection pump (RWD 68606, Shenzhen RWD). The injection needle was left in place for an additional 2 min to ensure complete reagent diffusion. Incisions were closed with disposable sterile sutures, and rats were closely monitored during the post‐operative recovery period.

For local DHHC2 knockdown, green fluorescent protein (GFP)‐tagged lentiviral (LV) vectors encoding short hairpin RNA (shRNA) targeting DHHC2 (LV‐DHHC2) were bilaterally injected into the LA of adult rats. For viral‐mediated overexpression of PSD‐95 or ABHD17B, GFP‐tagged LV vectors encoding rat PSD‐95 (LV‐PSD‐95) or ABHD17B (LV‐ABHD17B; GeneChem, Shanghai, China) were stereotaxically delivered into the LA (1.0 µL per side). Injection accuracy was verified via GFP immunohistochemistry (IHC). Behavioral testing commenced 4 weeks post‐injection for LV‐PSD‐95 and LV‐ABHD17B, and 2 weeks post‐injection for LV‐DHHC2. Following completion of behavioral testing, rats were anesthetized and transcardially perfused. Serial coronal brain sections were prepared for IHC analysis to confirm viral targeting.

### 4.5 Brain Slice Preparation

Male Sprague Dawley (SD) rats (7–8 weeks old) were anesthetized with isoflurane and transcardially perfused with ice‐cold cutting solution (4°C) composed of (in mm): 135 N‐methyl‐D‐glucamine, 1 KCl, 1.2 KH_2_PO_4_, 20 choline‐HCO_3_, 11 glucose, 0.5 CaCl_2_, 1.5 MgCl_2_ (pH adjusted to 7.4 with HCl, saturated with 95% O_2–_5% CO_2_). Following rapid decapitation, the brains were immediately transferred to ice‐cold, oxygenated cutting solution. Coronal brain slices (300 µm thick) containing the bilateral amygdala were sectioned using a vibratome (VT 1200S, Leica, Wetzlar, Germany) in cutting solution, then transferred to a holding chamber filled with artificial cerebrospinal fluid (aCSF) for incubation at 34°C for 30 min. Subsequently, slices were maintained at 28°C in aCSF continuously bubbled with 95% O_2–_5% CO_2_. The aCSF composition was (in mM): 119.0 NaCl, 1.3 MgSO_4_, 3.5 KCl, 1.0 NaH_2_PO_4_, 26.2 NaHCO_3_, 2.5 CaCl_2_, 11.0 glucose (pH 7.4, 300 mOsm).

### 4.6 Whole‐Cell Patch‐Clamp Recording

This method has been described before [80]. Following a 1.5‐h recovery period, brain slices were transferred to a recording chamber continuously perfused with oxygenated aCSF at a controlled flow rate of 2 mL/min. For recording AMPA receptors (AMPARs)‐mediated miniature excitatory postsynaptic currents (mEPSCs) from amygdalar neurons, patch electrodes (3–5 MΩ resistance) were used in a submersion chamber and filled with an internal solution composed of (in mM): 122.5 Cs‐gluconate, 17.5 CsCl, 0.2 EGTA, 10.0 HEPES, 1.0 MgCl_2_, 4.0 Mg‐ATP, 0.3 Na‐GTP, and 5.0 QX‐314 (pH adjusted to 7.2 with CsOH; 290–320 mOsm). Whole‐cell patch‐clamp recordings were performed using a Multiclamp 700B amplifier (Molecular Devices, Sunnyvale, CA, USA), with signals filtered at 3 kHz, amplified 5‐fold, and digitized at 20 kHz using a Digidata 1550A digitizer and Clampex 10 software (Molecular Devices, Sunnyvale, CA, USA). To isolate AMPAR‐mediated mEPSCs, recordings were conducted in bath solution supplemented with bicuculline (20 µm) and TTX (1 µM). All neurons were voltage‐clamped at −70 mV; recordings were excluded if series resistance changed by >20% or exceeded 15 MΩ, as monitored via a 10‐mV test pulse. The frequency and amplitude of mEPSCs were visually inspected and analyzed using the Mini Analysis Program (Synaptosoft, Decatur, GA, USA).

### 4.7 Field Potential Recording

The field potential recording was performed as described previously [81]. Following the same 1.5‐h recovery period, individual brain slices were transferred to a recording chamber continuously perfused with oxygenated aCSF at a flow rate of 2 mL/min. Field excitatory postsynaptic potentials (fEPSPs) evoked in cortical inputs to the lateral amygdala (LA) were recorded using glass recording electrodes (3–5 MΩ) filled with 3 M NaCl, positioned within the LA. Stimuli were delivered at 0.03 Hz to establish a stable baseline, with fEPSPs recorded for at least 20 min prior to long‐term potentiation (LTP) induction. LTP was induced via high‐frequency stimulation (HFS), consisting of three trains of 100 pulses (100 Hz) separated by 30 s, delivered at the test stimulus intensity. Post‐HFS fEPSP recording with single pulses was continued for 90 min to assess stable LTP expression. LTP data were acquired without concurrent recordings from a nontetanized control pathway. fEPSP slopes were normalized to the average slope during the pre‐HFS baseline period, and all LTP data were quantified as the average slope during the final 15 min of the 90‐min post‐HFS recording period.

### 4.8 Primary Hippocampal Neuron Culture

Hippocampi were dissected from postnatal day 0 (P0) rat pups. Following enzymatic dissociation using papain, cells were plated onto poly‐D‐lysine‐coated culture surfaces. Neurons were cultured in Neurobasal medium supplemented with B‐27 Supplement and GlutaMAX. To suppress glial proliferation, 5 µm cytosine arabinoside (Ara‐C) was added on day 3 in vitro (DIV 3). Cultures were maintained at 37°C in a humidified atmosphere of 5% CO_2_, with half the medium refreshed every 3 days.

### 4.9 Chemical LTP Induction in Cultured Hippocampal Neurons

Previous studies have a similar paradigm [82, 83]. Following pre‐incubation in aCSF at 37°C, neurons were treated with aCSF supplemented with 1 µm TTX at 37°C for 5 min. The solution was then aspirated and replaced with Mg^2^^+^‐free aCSF containing 200 µm glycine, and incubation continued at 37°C for another 5 min. This was followed by a subsequent recovery in Mg^2^^+^‐containing aCSF supplemented with TTX.

### 4.10 Western Blotting

LA tissues were carefully dissected under a dissecting microscope using a sterile scalpel. RIPA lysis buffer [50 mm Tris‐HCl (pH 7.4), 1% NP‐40, 0.5% sodium deoxycholate, 0.1% sodium dodecyl sulfate (SDS), 2 mm EDTA, 50 mm NaF, 150 mm NaCl] supplemented With phosphatase inhibitors (Sigma‐Aldrich, St. Louis, MO, USA) and a protease inhibitor cocktail (Roche, Basel, Switzerland) was added at a ratio of 10 µL per 1 mg of tissue. The tissue was lysed on ice for 20–30 min, homogenized, and centrifuged at 12 000 × g for 20 min at 4°C. The protein concentration of the supernatant was quantified using a Coomassie Brilliant Blue (CBB) protein assay kit (Nanjing Jiancheng Institute of Biological Engineering, Nanjing, China), with bovine serum albumin (BSA) as the standard. Samples were heated at 95°C for 5 min in SDS loading buffer, loaded Onto 10% SDS‐polyacrylamide gel electrophoresis (SDS‐PAGE) gels, and separated by electrophoresis. Proteins were then transferred to nitrocellulose membranes using transfer buffer [25 mm Tris, 190 mm glycine, 20% methanol, 0.5% SDS (pH 8.3)]. Membranes were blocked in Tris‐buffered saline with Tween‐20 (TBST; 50 mM Tris‐HCl, pH 7.4, 150 mM NaCl, 0.5% Tween‐20) containing 5% (w/v) BSA for 1 h at room temperature, followed by overnight incubation with primary antibodies at 4°C. After three 10‐min washes with TBST, membranes were incubated with horseradish peroxidase (HRP)‐conjugated secondary antibodies for 1 h at room temperature. Membranes were rinsed with double‐distilled water (ddH_2_O) and incubated with enhanced chemiluminescence (ECL) substrate (SuperSignal West Pico; Pierce Chemical Co., Rockford, IL, USA). Immunoreactive bands were visualized using a MicroChemi imaging system (DNR Bio‐imaging Systems, Jerusalem, Israel). Band optical densities were quantified using NIH ImageJ software. All results were normalized to the control group and expressed as a percentage of control. Detailed information about the antibodies used is provided in Table S1.

### 4.11 Acyl‐Biotin Exchange (ABE) Assay

This method has been described in our previous study [84, 85]. Ice‐cold RIPA lysis buffer [50 mM Tris‐HCl (pH 7.4), 150 mm NaCl, 0.5% sodium deoxycholate, 1% NP‐40, 2% sodium dodecyl sulfate (SDS), 1 mM ethylene diamine tetraacetate (EDTA)] supplemented with a protease inhibitor cocktail and 20 mm S‐methyl methanethiosulfonate (MMTS; Sigma‐Aldrich, St. Louis, MO, USA) was added to dissected LA tissues to block free thiols. Tissues were homogenized by sonication (10 s) and incubated on ice for 30 min to allow MMTS reaction, followed by centrifugation at 12 000 × g for 20 min at 4°C. The supernatant was collected, and four volumes of blocking buffer [1 volume 25% SDS + 9 volumes HEN buffer (250 mm Hepes‐NaOH, pH 7.7; 1 mm EDTA; 1 mm neocuproine) supplemented with 20 mm MMTS] were added to each sample. Samples were mixed thoroughly, incubated at 50°C in the dark with gentle shaking for 30 min to complete free thiol blocking. Residual MMTS was removed by adding four volumes of cold acetone, incubating at −20°C for 30 min, and centrifuging at 12 000 × g for 20 min. The pellet was collected, resuspended in RIPA buffer, and this acetone precipitation step was repeated twice.

The final pellet was resuspended in RIPA buffer and split into two equal aliquots: (1) Control group: incubated with HPDP‐biotin (Thermo Fisher Scientific, Waltham, MA, USA) in RIPA buffer (without hydroxylamine, HA); (2) Palmitoylation detection group: incubated with four volumes of RIPA buffer containing 0.7 mm HA (Sigma‐Aldrich, St. Louis, MO, USA) to cleave thioester bonds and 1 mm HPDP‐biotin to biotinylate newly exposed cysteine thiols. All samples were incubated at room temperature in the dark for 2 h with frequent vortexing. Remaining HPDP‐biotin and HA were removed by acetone precipitation, and the pellet was resuspended in RIPA buffer. Protein concentration was quantified using a Coomassie Brilliant Blue (CBB) protein assay kit (Nanjing Jiancheng Institute of Biological Engineering, Nanjing, China).

For global palmitoylation detection, samples were subjected to Western blot analysis using horseradish peroxidase (HRP)‐conjugated streptavidin. For targeted palmitoylation analysis, each ABE‐treated sample was split into two portions: (1) “Input” fraction: directly mixed with SDS‐PAGE loading buffer; (2) Affinity‐purified fraction: incubated with streptavidin‐agarose beads (Sigma‐Aldrich, St. Louis, MO, USA) for 10 h at 4°C with gentle rotation. Beads were washed three times with RIPA buffer to remove non‐specifically bound proteins, then eluted and denatured in SDS‐PAGE loading buffer at 95°C for 5 min. Biotinylated samples were stored at −80°C until Western blot analysis. Palmitoylated protein levels were normalized to the corresponding input (total protein) and calibrated to β‐actin as an internal reference.

### 4.12 RT‐qPCR

Relative mRNA levels of DHHC2 and ABHD17A/B/C were quantified using a Real‐Time PCR System (Applied Biosystems, Foster City, CA, USA) with the SYBR Green Premix Ex Taq kit (Takara, Shiga, Japan). Total RNA was isolated from LA tissues using TRIzol reagent (Invitrogen, Carlsbad, CA, USA). Reverse transcription (RT) was performed with 4 µL of 5× miScript Reaction Mix and 1 µL of miScript Reverse Transcriptase (QIAGEN, Hilden, Germany; note: MiScript reagents are typically used for miRNA, adjusted to align with mRNA detection context unless specified otherwise). PCR reactions were carried out in a total volume of 10 µL, containing 2 µL of cDNA template, 0.6 µL of primer mix (forward + reverse), 5 µL of SYBR Green Premix Ex Taq, and 2.4 µL of RNase‐free water. Blank controls (no template) were included in triplicate for each master mix. The thermal cycling conditions were as follows: Initial denaturation at 95°C for 30 s, followed by 40 cycles of denaturation at 95°C for 5 s, primer annealing at 60°C for 30 s, and extension at 72°C for 30 s. After amplification, a melting curve analysis was performed (60°C to 95°C, with a 0.5°C increment every 10 s) to verify amplicon specificity. Primers for target genes were purchased from GeneChem (Shanghai, China). Relative transcript levels were normalized to the housekeeping gene GAPDH. All experiments were performed in duplicate. Detailed information of the primers used is provided in Table S2.

### 4.13 Co‐Immunoprecipitation (Co‐IP) Assay

Co‐IP assay was performed as previously reported [61]. Tissue samples were lysed in 0.2 mL of Co‐IP lysis buffer [150 mm NaCl, 0.2% NP‐40, 50 mm NaF, 1 mM Na_3_VO_4_, 6 mm sodium deoxycholate, 3 mM sodium pyrophosphate, 1× protease inhibitor cocktail, 1× phosphatase inhibitor cocktail] and incubated on ice for 30 min with gentle agitation to ensure complete lysis. Lysates were centrifuged at 12 000 × g for 15 min at 4°C, and 500 µg of total protein from the supernatant was collected for subsequent experiments. The protein extract was incubated with 2 µg of anti‐PSD‐95 antibody (detailed information in Table S1) at 4°C overnight with gentle rotation.

The antibody‐antigen complexes were captured by adding 50 µL of pre‐equilibrated Protein A/G PLUS‐Agarose beads (Santa Cruz Biotechnology, Dallas, TX, USA) and incubating at 4°C for 3–4 h with gentle rotation. Beads were collected by centrifugation at 4 000 × g for 3 min and washed three times with wash buffer [10 mm Hepes (pH 7.5), 100 mm NaCl, 1 mm EDTA, 10% glycerol, 0.1% Triton X‐100] to remove non‐specifically bound proteins. Finally, immunoprecipitated proteins were eluted from the beads with 50 µL of SDS‐PAGE loading buffer and boiled at 95°C for 5 min to denature proteins. The eluted samples were analyzed by Western blot as described above.

### 4.14 Postsynaptic Density (PSD) Purification

PSD purification assay was performed as described previously [86]. Tissue samples were homogenized by sonication on ice in ice‐cold TEVP lysis buffer [0.32 mm sucrose, 10 mM Tris‐HCl (pH 7.4), 5 mm NaF, 1 mm Na_3_VO_4_, 1 mM EDTA, 1 mm EGTA, 1× protease and phosphatase inhibitor cocktail]. Homogenates were centrifuged at 1 000 × g for 10 min at 4°C to pellet nuclei and large debris, and the supernatant was collected. This supernatant was then centrifuged at 10 000 × g for 30 min at 4°C to obtain the crude synaptosomal pellet (the resulting supernatant was discarded). The crude synaptosomal pellet was resuspended and subjected to discontinuous sucrose density gradient centrifugation (0.85/1.0/1.2 M sucrose) at 100 000 × g for 2 h at 4°C. Synaptosomes were collected from the 1.0/1.2 M sucrose interface. The synaptosomal fraction was washed with ice‐cold 0.5% Triton X‐100 in TEVP buffer, then centrifuged at 1 000 × g for 10 min at 4°C to collect the synaptosomal pellet. This pellet was resuspended and subjected to a second discontinuous sucrose density gradient centrifugation (1.0/1.5/2.0 M sucrose) at 100,000 × g for 2 h at 4°C. The PSD fraction was isolated from the 1.5/2.0 M sucrose interface. The PSD fraction was diluted with an equal volume of ice‐cold 1% Triton X‐100/150 mM KCl solution, vortexed gently for 5 min, and centrifuged at 201,800 × g for 1 h at 4°C. The final PSD pellet was resuspended in ice‐cold 0.5% Triton X‐100/75 mm KCl solution supplemented with sodium orthovanadate and protease inhibitors. All procedures were performed at 4°C to prevent protein degradation.

### 4.15 Surface Biotinylation Assay

This assay was performed as our previous study [87]. Tissue samples were collected into Eppendorf tubes containing ice‐cold aCSF [119.0 mm NaCl, 1.3 mm MgSO_4_, 3.5 mM KCl, 1.0 mM NaH_2_PO_4_, 26.2 mm NaHCO_3_, 2.5 mM CaCl_2_, 11.0 mm glucose (pH 7.4, 300 mOsm)] supplemented with 1 mg/mL EZ‐Link Sulfo‐NHS‐LC Biotin (Pierce Biotechnology, Rockford, IL, USA). Samples were incubated at 4°C for 2 h with gentle shaking to allow surface protein biotinylation. The reaction was quenched with 100 mm glycine on ice for 30 min to terminate biotinylation, followed by three washes with ice‐cold aCSF to remove unreacted biotin.

Tissues were homogenized in RIPA lysis buffer (10 µL per 1 mg tissue) [50 mM Tris‐HCl (pH 7.4), 1% NP‐40, 0.5% sodium deoxycholate, 0.1% SDS, 150 mm NaCl, 2 mM EDTA, 50 mm NaF] supplemented with a protease and phosphatase inhibitor cocktail (Sigma‐Aldrich). Lysates were centrifuged at 12 000 × g for 15 min at 4°C, and the supernatant was collected. Protein concentration in the supernatant was quantified using a Coomassie Brilliant Blue (CBB) protein assay kit (Nanjing Jiancheng Institute of Biological Engineering, Nanjing, China). A total of 200–300 µg of biotinylated protein from the supernatant was incubated with streptavidin‐agarose beads (Pierce Biotechnology, Rockford, IL, USA) at 4°C for 4 h with gentle rotation. Streptavidin‐bound protein complexes were washed three times with RIPA lysis buffer to remove non‐specifically bound proteins, with each wash followed by centrifugation at 4 000 × g for 3 min at 4°C. Bound proteins were eluted from the beads and denatured in SDS‐PAGE loading buffer at 95°C for 10 min prior to Western blot analysis.

### 4.16 Tissue Immunofluorescence Assay

Adult male SD rats were anesthetized with tribromethanol (250 mg/kg, intraperitoneal injection [i.p.]) and transcardially perfused with ice‐cold phosphate‐buffered saline (PBS), followed by 4% paraformaldehyde (PFA) in PBS. Brains were post‐fixed overnight at 4°C, then cryoprotected in graded sucrose solutions (20%, followed by 30% in PBS) at 4°C. Tissues were embedded in optimal cutting temperature (O.C.T.) compound and cryosectioned into 10‐µm coronal sections.

For immunostaining, sections were subjected to antigen retrieval in 10 mm citrate buffer (pH 6.0) at 95°C for 15 min, followed by cooling to room temperature (RT) naturally. Sections were permeabilized with 0.1% Triton X‐100 in PBS for 15 min at RT, then blocked in Immunostaining Blocking Buffer (Beyotime, P0102) for 2 h at RT. Primary antibodies, diluted in Primary Antibody Dilution Buffer (Beyotime, P0103), were incubated with sections overnight at 4°C. After three washes with PBS (5 min per wash), sections were incubated with fluorochrome‐conjugated secondary antibodies for 4 h at RT in the dark. Nuclei were counterstained with 4',6‐diamidino‐2‐phenylindole (DAPI) for 4 min at RT, and sections were mounted with anti‐fade mounting medium. Fluorescent images were acquired using a confocal microscope with identical acquisition settings. Mean fluorescence intensity (MFI) was quantified using ImageJ/Fiji software. Detailed information of the antibodies used is provided in Table S1.

### 4.17 Cell Immunofluorescence Assay

Primary hippocampal neurons were cultured on poly‐D‐lysine‐coated coverslips. At 14 days in vitro (DIV 14), cells were fixed With 4% paraformaldehyde (PFA) in phosphate‐buffered saline (PBS) for 15 min at room temperature (RT), followed by three washes With PBS (5 min per wash).

For immunostaining, cells were permeabilized With 0.1% Triton X‐100 in PBS for 15 min at RT, then blocked in Immunostaining Blocking Buffer (Beyotime, P0102; consistent With tissue immunofluorescence reagents) for 2 h at RT. Primary antibodies, diluted in Primary Antibody Dilution Buffer (Beyotime, P0103), were incubated with cells overnight at 4°C. After three washes with PBS (5 min per wash), cells were incubated with fluorophore‐conjugated secondary antibodies for 4 h at RT in the dark. Nuclei were counterstained with 4',6‐diamidino‐2‐phenylindole (DAPI) for 4 min at RT. Coverslips were mounted onto glass slides using anti‐fade mounting medium. Fluorescent images were acquired with identical acquisition settings using a confocal microscope. Mean fluorescence intensity (MFI) was quantified using ImageJ/Fiji software. Detailed information of the antibodies used is provided in Table S1.

### 4.18 Molecular Docking Analysis

Protein structures were derived from either experimentally resolved crystal structures (PDB accession codes provided in Table S3) or AlphaFold2‐predicted models (UniProt IDs listed in Table S3). All structures were processed using PyMOL software (Schrödinger, LLC) to remove solvent molecules, water, and non‐protein heteroatoms (e.g., ligands, ions). The refined protein structures were assembled into a protein–protein complex, with each chain assigned a unique identifier to distinguish individual subunits. For the membrane‐associated protein, only the cytosolic functional region was retained to avoid interference from transmembrane helices. Protein–protein docking was performed using the ZDOCK 3.0.2 (Weng lab) to generate plausible binding conformations. Docked structures were evaluated by RMSD‐based clustering, and a representative structure from the most populated cluster was selected for subsequent analysis. Binding free energy of the protein–protein complex was estimated using the PRODIGY web server based on interfacial contacts [88]. Binding affinity (ΔG) and dissociation constant (K_d_) were calculated at 25°C, and interface contact composition was analyzed to characterize the physicochemical nature of the interaction.

### 4.19 Statistical Analysis

All statistical analyses were performed using SPSS 18.0 software (SPSS Inc., Chicago, IL, USA) or GraphPad Prism 9 (GraphPad Software, San Diego, CA, USA). Data are presented as mean ± standard error of the mean (SEM). Sample sizes were estimated a priori based on previous studies or pilot experiments to ensure adequate statistical power. Prior to parametric analyses, normality of data distribution was verified using the Shapiro–Wilk test, and homogeneity of variance was assessed with Levene's test.

Statistical comparisons were performed using two‐tailed unpaired Student's *t*‐test for two independent groups, one‐way analysis of variance (ANOVA) for multiple groups with a single independent variable, or two‐way ANOVA for groups with two independent variables, as dictated by the experimental design. Post hoc analyses for significant ANOVA results were conducted using Tukey's honest significant difference (HSD) test to correct for multiple comparisons. A two‐tailed p‐value < 0.05 was considered statistically significant. Detailed statistical parameters (e.g., test type, degrees of freedom, F/t values, p‐values) and sample sizes (n) for each experiment are provided in the corresponding figure legends.

## Author Contributions

Z‐C.S. conducted the majority of the experiments and drafted the manuscript. Y.‐X.W. performed stereotaxic brain microinjections and behavioral tests. Z.‐X.X. acquired and analyzed electrophysiological recording data. Y.‐L.Z. assisted with experimental methodology and data analysis. Q.‐Q.D. and S.‐Y.W. assisted with tissue collection. T.C. and X.‐Y.C. contributed to immunostaining experiments and data analysis. W.‐C.T. participated in project discussions. S.L. and Z.C. contributed to project discussions and critically reviewed the manuscript. Z.‐C.S. and Y.‐X.L. conceived and designed the study, supervised the research, and revised the manuscript. All authors read and approved the final manuscript.

## Conflicts of Interest

The authors declare no conflicts of interest.

## Supporting information




**Supporting File**: advs76302‐sup‐0001‐SuppMat.doc.

## Data Availability

The data that support the findings of this study are available from the corresponding author upon reasonable request.
