# Supplementary material for: Palmitoylation of PSD‐95 Orchestrates Learning‐Dependent Metaplasticity in the Amygdala and Fear Memory
Source: Adv Sci (Weinh). 2026 Jul 6:e76302. Online ahead of print. doi: 10.1002/advs.76302 (PMC13335701; doi:10.1002/advs.76302)
Supplement: Supplementary file 1 — Supporting File: advs76302‐sup‐0001‐SuppMat.doc. [file ADVS-9999-e76302-s001.doc]

**Supplemental Material for**

**Palmitoylation of PSD‑95 orchestrates learning‑dependent metaplasticity in the amygdala and fear memory formation**

Zu-Cheng Shen^1,#^,*, Yu-Xuan Weng^2,#^, Zhi-Xuan Xia^3,#^, Yue-Ling Zhao^1,#^, Qian-Qian Ding^1^, Si-Ying Wang^1^, Ting Cao^4^, Xuan-Ying Chen^2^, Wu-Cheng Tao^2^, Shen Lin^5^,*, Zhou Chen^6^,*, Yi-Xiao Luo^7,8^,*

^1^Department of Pharmacology, School of Pharmacy, Fujian Medical University, Fuzhou, 350122, China;

^2^Key Laboratory of Brain Aging and Neurodegenerative Diseases, Fujian Medical University, Fuzhou, 350122, China;

^3^Key Laboratory of Tropical Translational Medicine of Ministry of Education, School of Basic Medicine and Life Sciences, Hainan Medical University, Haikou, 571199, China;

^4^Department of Stomatology, Union Hospital, Fujian Medical University, Fuzhou, 350001, China;

^5^Fujian Provincial Institutes of Brain Disorders and Brain Sciences, First Affiliated Hospital, Fujian Medical University, Fuzhou, 350004, China;

^6^Department of Clinical Pharmacy and Pharmacy Administration, School of Pharmacy, Fujian Medical University, Fuzhou, 350122, China;

^7^Beijing Key Laboratory of Intelligent Drug Research and Development for Mental Disorders; National Clinical Research Center for Mental Disorders; National Center for Mental Disorders; Beijing Anding Hospital, Capital Medical University, Beijing, 100088, China;

^8^School of Pharmaceutical Sciences, Hunan Normal University, Changsha, 410013, China;

^#^These authors contributed equally to this work;

*Correspondence: [shenzc2019fjmu@163.com](mailto:shenzc2019fjmu@163.com) (Z.-C.S.), 0621133@zju.edu.cn (S.L.), chenzhou@fjmu.edu.cn (Z.C.) and Luoyx@hunnu.edu.cn (Y.-X.L.).

**Supplemental Figures and legends**


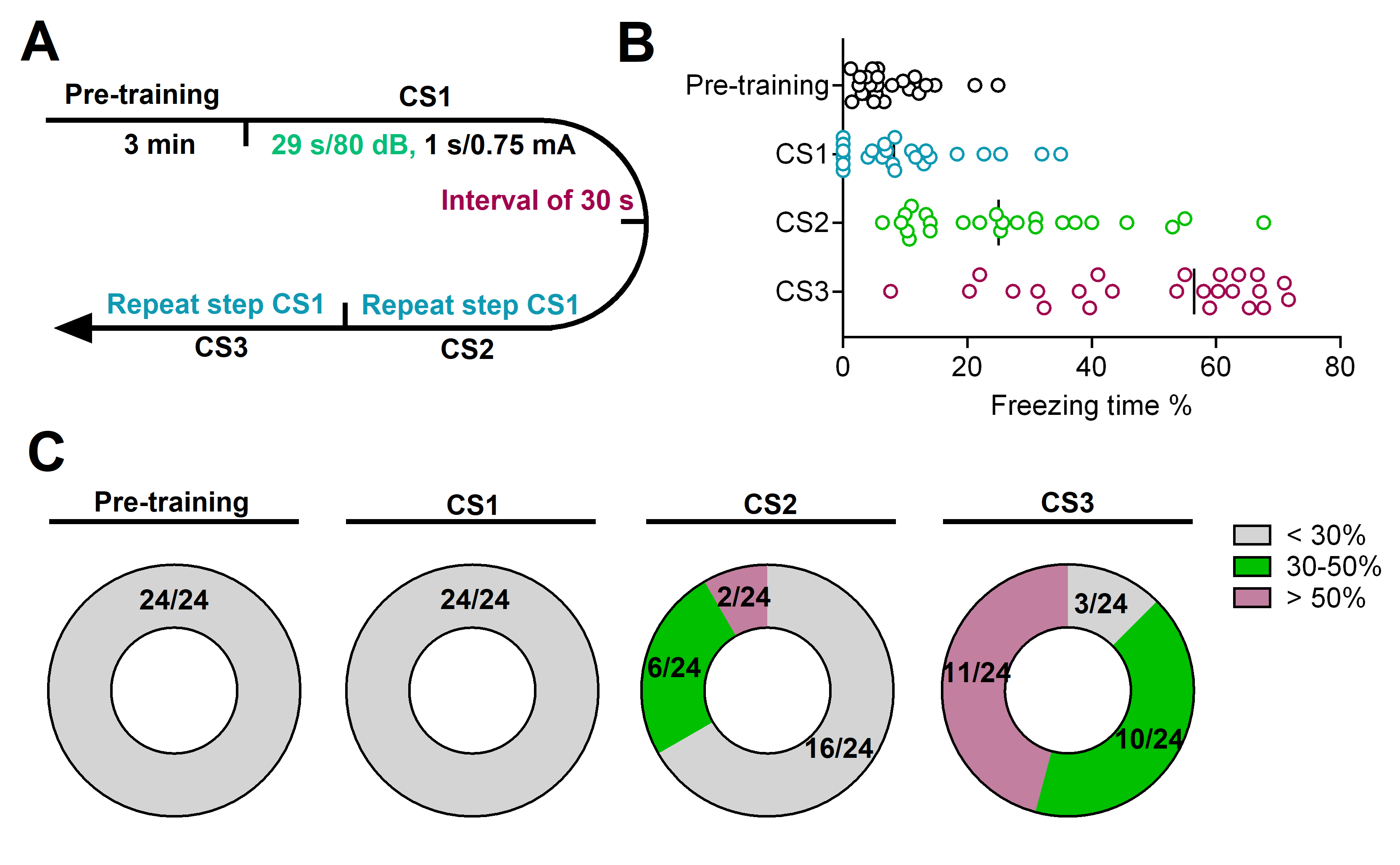


**Figure S1. Cue fear conditioning in male rats.** (**A**) Schematic timeline of the cue fear conditioning paradigm. (**B**) Percentage of freezing time during pre-training and conditioned stimulus (CS) presentations 1–3. (**C**) Distribution of animals across different freezing level categories during pre-training and CS presentations 1–3 (n = 24 rats per group).


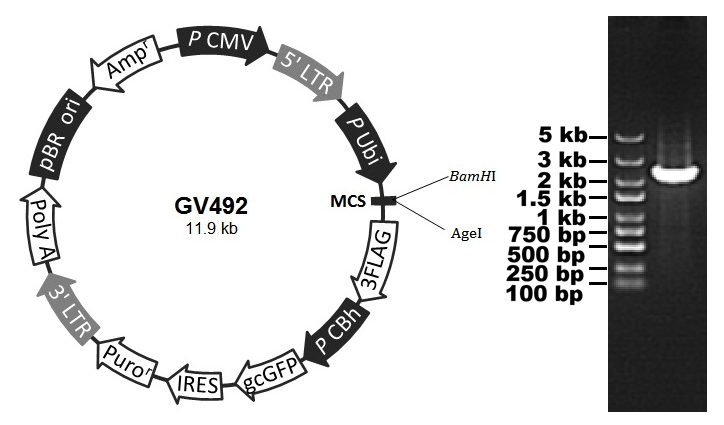


**Figure S2. Construction of PSD-95 overexpression lentiviral vectors.** Left panel shows the order of elements, and the right panel shows the PCR results (Size of the PCR product: 2216 bp).


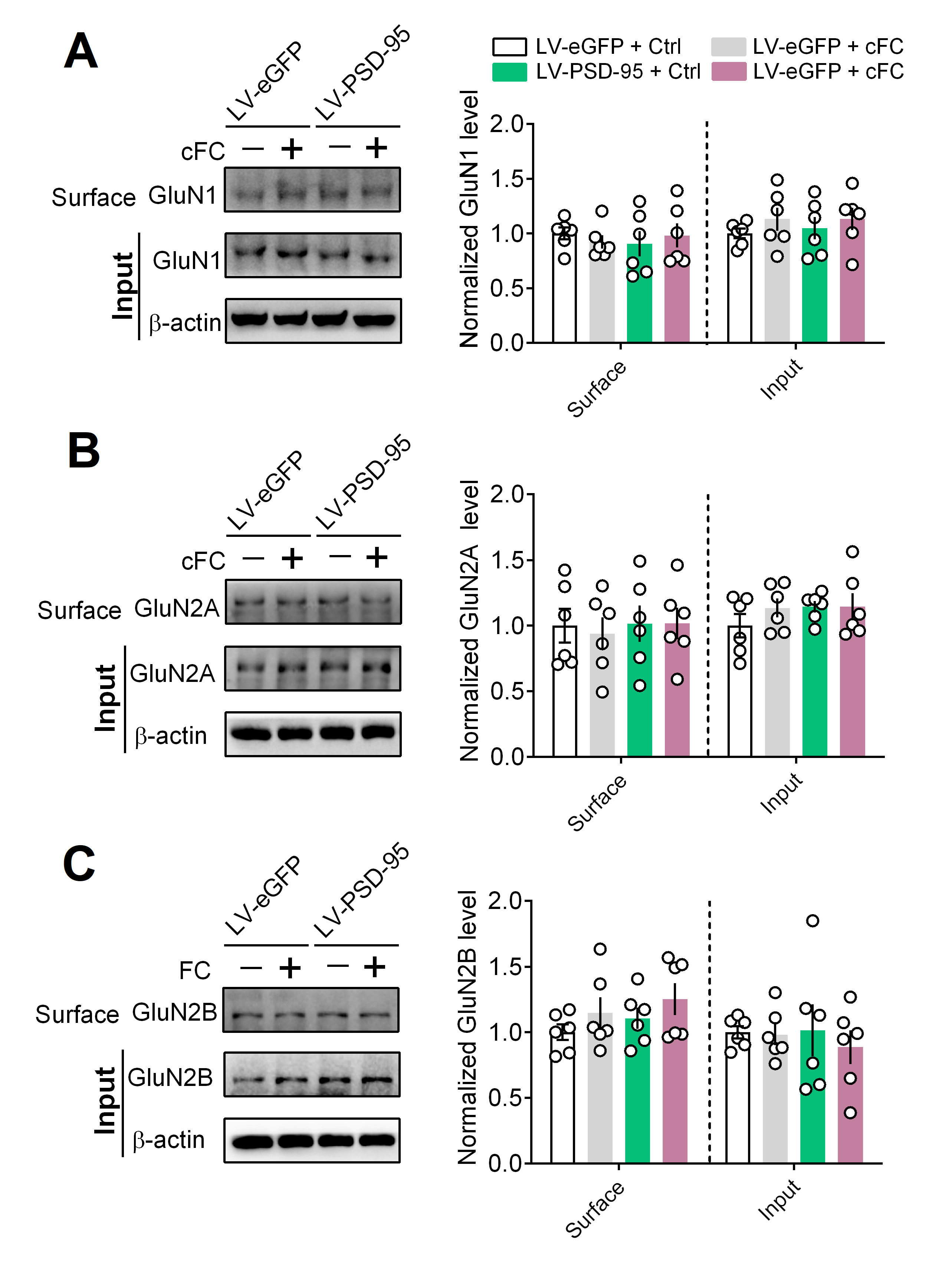


**Figure S3. Effects of PSD-95 overexpression on NMDA receptor subunits and their surface expression in the LA.** (**A** to **C**) Representative Western blots (left) and quantitative analysis (right) of NMDA receptor subunits (GluN1, GluN2A, GluN2B) and their surface expression levels in the LA of rats subjected to PSD-95 overexpression and/or cue fear conditioning: (A) GluN1, (B) GluN2A, (C) GluN2B (n = 6 rats per group). Data are represented as mean ± SEM. Statistical analysis was performed using unpaired two-tailed Student’s t-test.

**
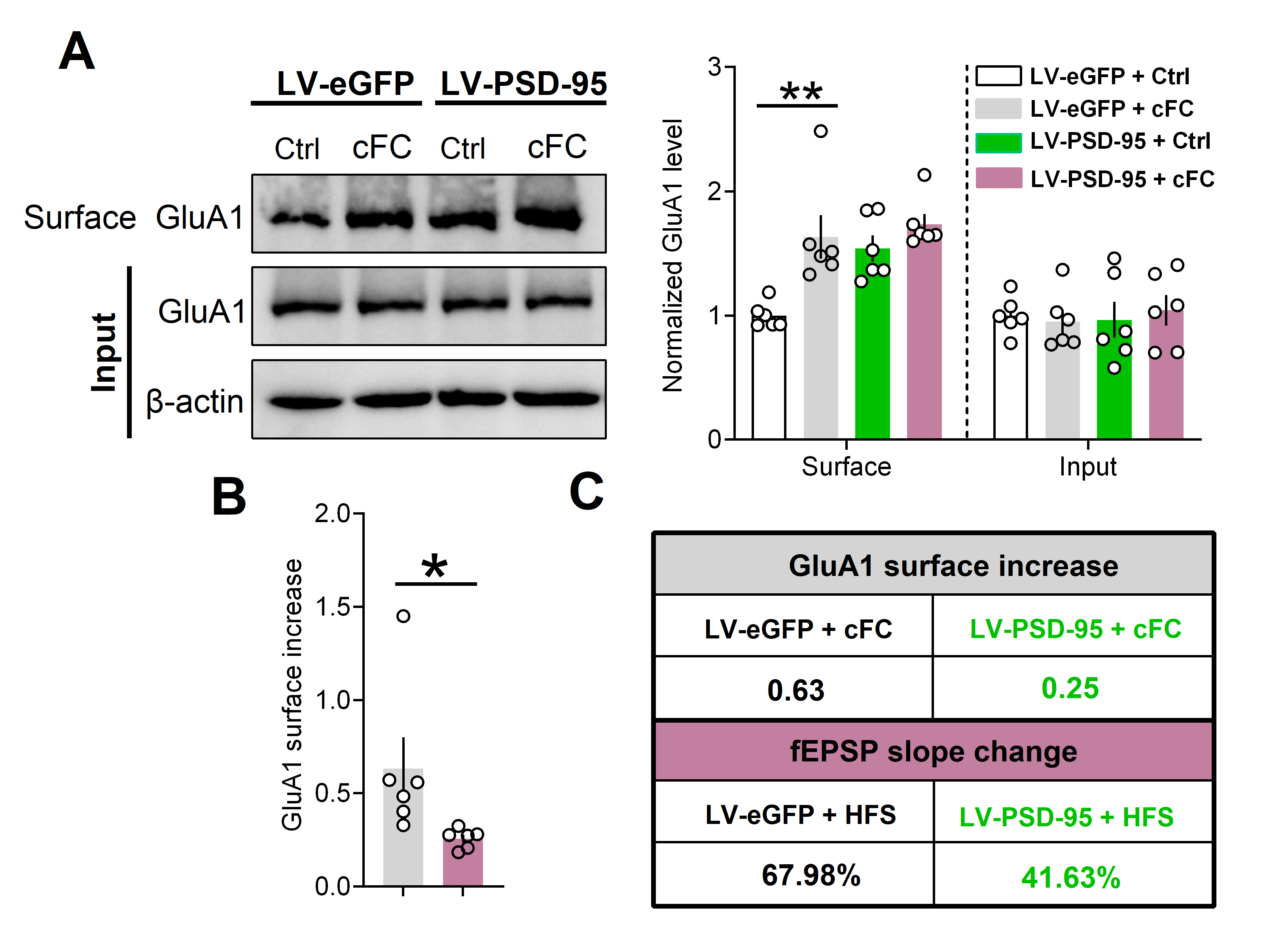
Figure S4. PSD-95 overexpression attenuates cFC-induced increase in surface GluA1 expression.** (**A**) Representative Western blots (left) and quantitative analysis (right) of GluA1 total and surface levels in the LA of rats with PSD-95 overexpression and/or cue fear conditioning (n = 6 rats per group). (**B**) Magnitude of cFC-induced surface GluA1 increase in the LA of rats with PSD-95 overexpression and/or cFC (n = 6 rats per group). (**C**) Magnitude of surface GluA1 increase and HFS-induced fEPSP slope change in rats with PSD-95 overexpression and/or cFC. Data are presented as mean ± SEM. *p < 0.05, ***p < 0.001. Statistical analyses were performed using unpaired two-tailed Student’s t-test (A) and one-way ANOVA followed by Tukey’s post hoc test (B).


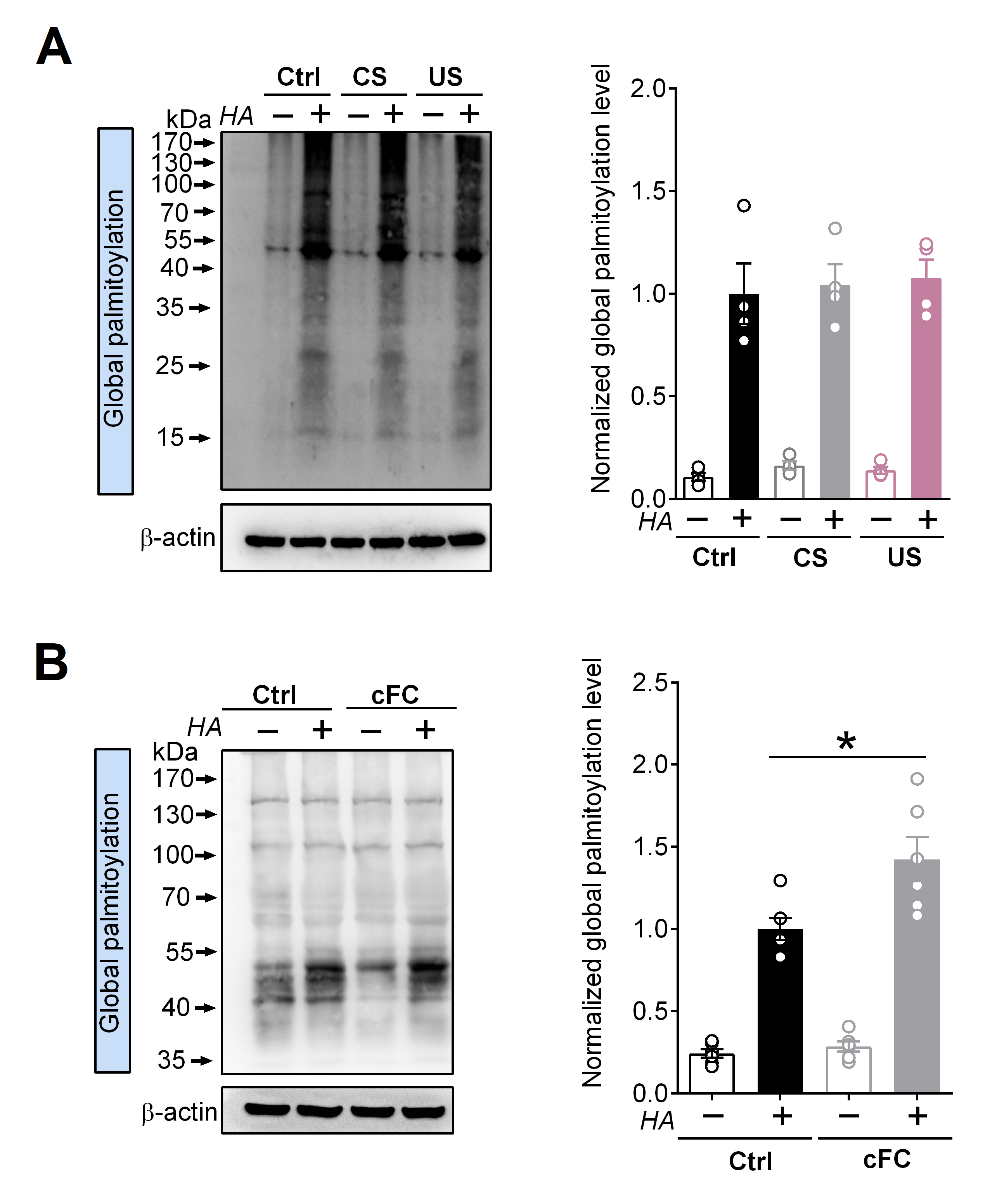


**Figure S5. Global palmitoylation in the LA.** (**A**) Representative Western blots (left) and statistical results (right) of streptavidin-labeled proteins in the LA of rats subjected to control (Ctrl), conditioned stimulus (CS) alone, or unconditioned stimulus (US) alone (n = 4 rats per group). (**B**) Representative Western blots (left) and statistical results (right) of streptavidin-labeled proteins in the LA of Ctrl and paired fear conditioning groups (n = 4-6 rats per group). Data are represented as mean ± SEM. *p < 0.05. Statistical analyses were performed using one-way ANOVA followed by Tukey’ s post hoc test (B).


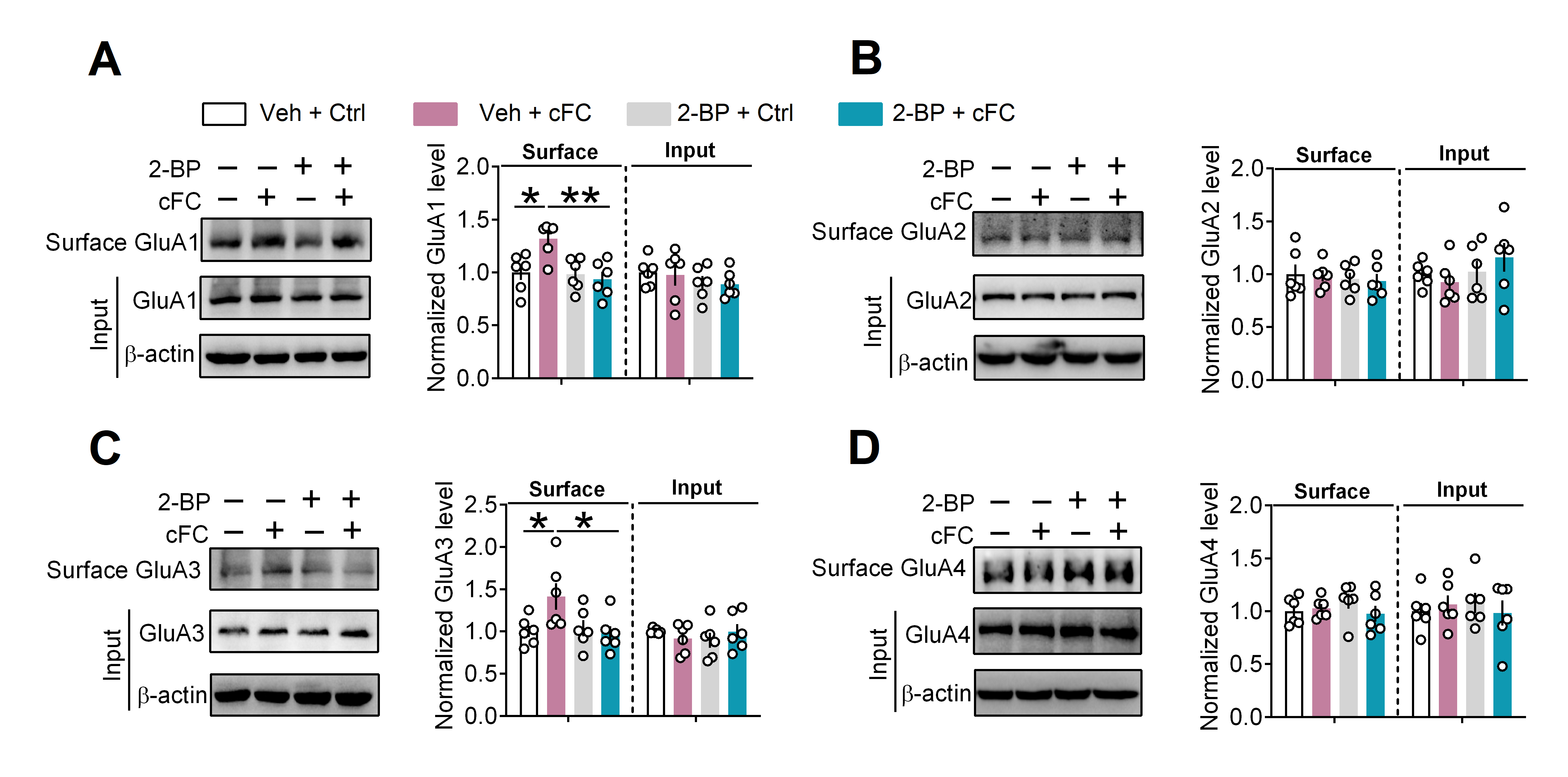


**Figure S6. Effects of 2-BP on GluA1–4 subunits in the cFC model.** (**A** to **D**) Representative Western blots (left) and quantitative analysis (right) of total and surface expression levels of AMPARs subunits in the LA from groups subjected to cue fear conditioning and/or 2-BP treatment: (A) GluA1, (B) GluA2, (C) GluA3, (D) GluA4 (n = 6 rats per group). Data are represented as mean ± SEM. *p < 0.05, **p < 0.01. Statistical analyses were performed using unpaired two-tailed Student’s t-test.


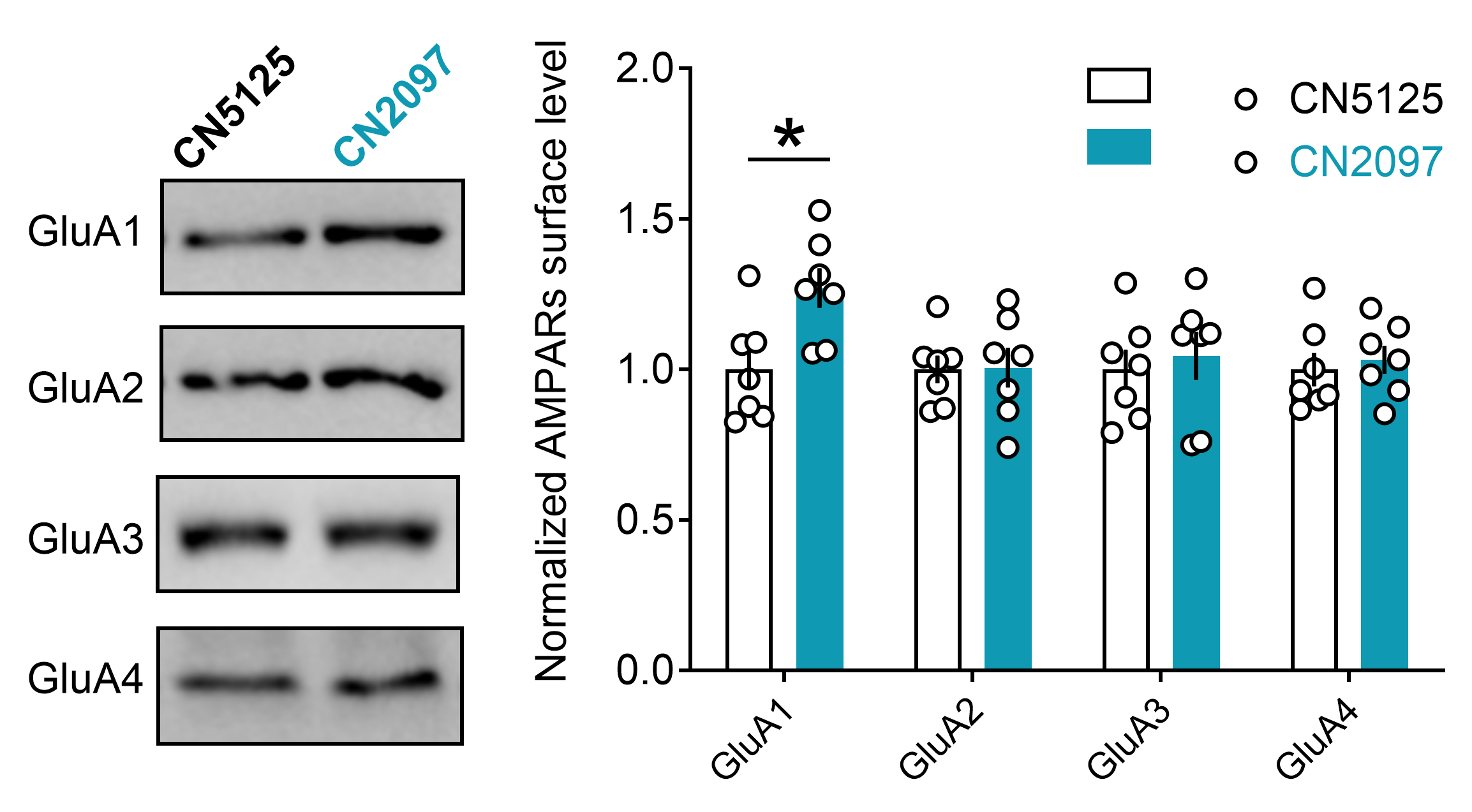


**Figure S7. GluA1-4 surface expression in the LA following CN2097 treatment.** Representative Western blots (left) and quantitative analysis (right) of GluA1–4 surface expression in the LA after CN2097 treatment (n = 7 rats per group). Data are presented as mean ± SEM. *p < 0.05. Statistical analyses were performed using one-way ANOVA followed by Tukey’s post hoc test.


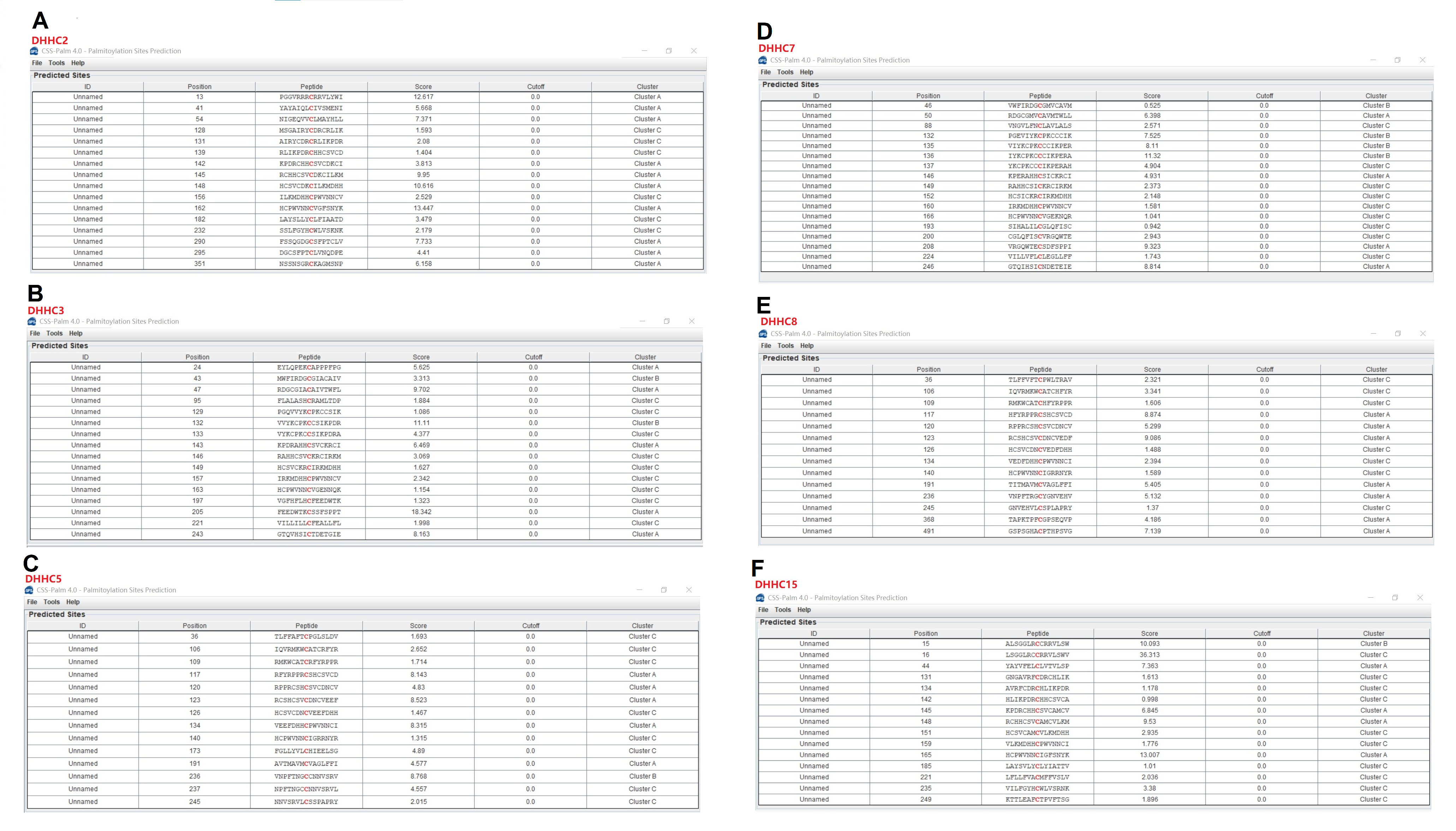


**Figure S8. Palmitoylation sites of DHHC proteins interacting with AMPARs and PSD-95.** Palmitoylation sites were predicted for (**A**) DHHC2, (**B**) DHHC3, (**C**) DHHC5, (**D**) DHHC7, (**E**) DHHC8, and (**F**) DHHC15 using CSS-Palm 4.0 software (CSS-Palm 4.0; [http://csspalm.biocuckoo.org](http://csspalm.biocuckoo.org/)).


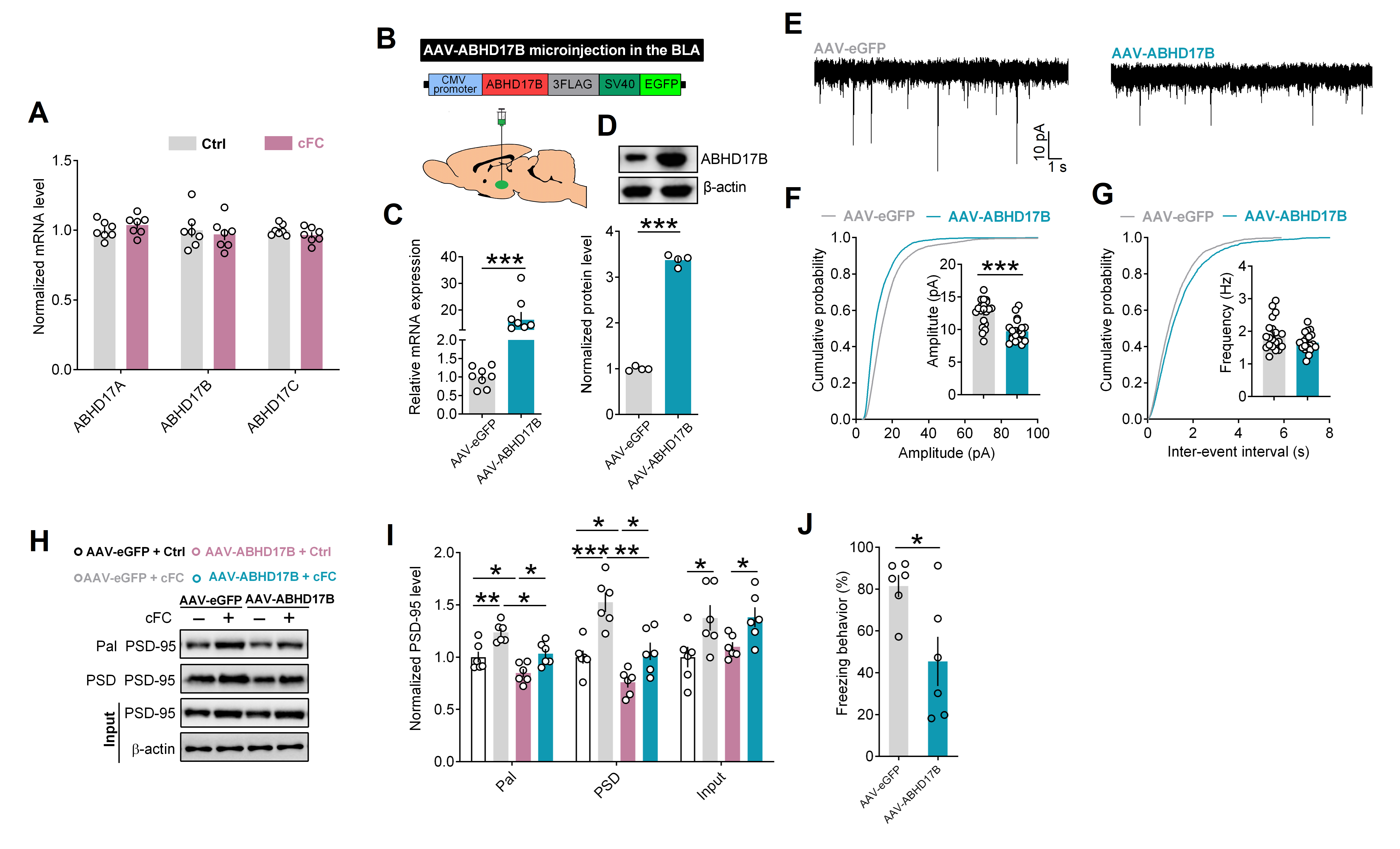


**Figure S9. Effects of ABHD17B overexpression on synaptic transmission in the LA.** (**A**) RT-qPCR analysis of ABHD17 subunits (ABHD17A/B/C) in the LA of rats after cue fear conditioning (n = 7 rats per group). (**B**) Schematic diagram showing the construction of AAV-ABHD17B vector and the stereotaxic injection procedure targeting the LA region. (**C**) Quantification of ABHD17B mRNA levels by RT-qPCR in the LA of rats injected with AAV-eGFP or AAV-ABHD17B (n = 7-8 rats per group). (**D**) Representative Western blots (top) and quantitative analysis (bottom) of ABHD17B protein expression in the LA of AAV-eGFP- and AAV-ABHD17B-injected rats (n = 4 rats per group). (**E**) Representative traces of mEPSCs recorded from LA neurons of AAV-eGFP- and AAV-ABHD17B-injected rats. (**F** and **G**) Cumulative probability distributions of mEPSCs frequency (F) and amplitude (G), along with their average values (insets) (n = 20 cells from 8 rats). (**H** and **I**) Representative Western blots (H) and quantitative analysis (I) of total PSD-95 expression, PSD-95 palmitoylation level, and PSD-enriched PSD-95 expression in the LA (n = 6 rats per group). (**J**) Cue fear memory test on day 3 after conditioning in rats with ABHD17B overexpression in the LA (n = 6 rats per group). Data are represented as mean ± SEM. *p < 0.05, **p < 0.01, ***p < 0.001. Statistical analyses were performed using unpaired two-tailed Student’s t test (I) and one-way ANOVA followed by Tukey’ s post hoc test (A, C, D, F and J).

**
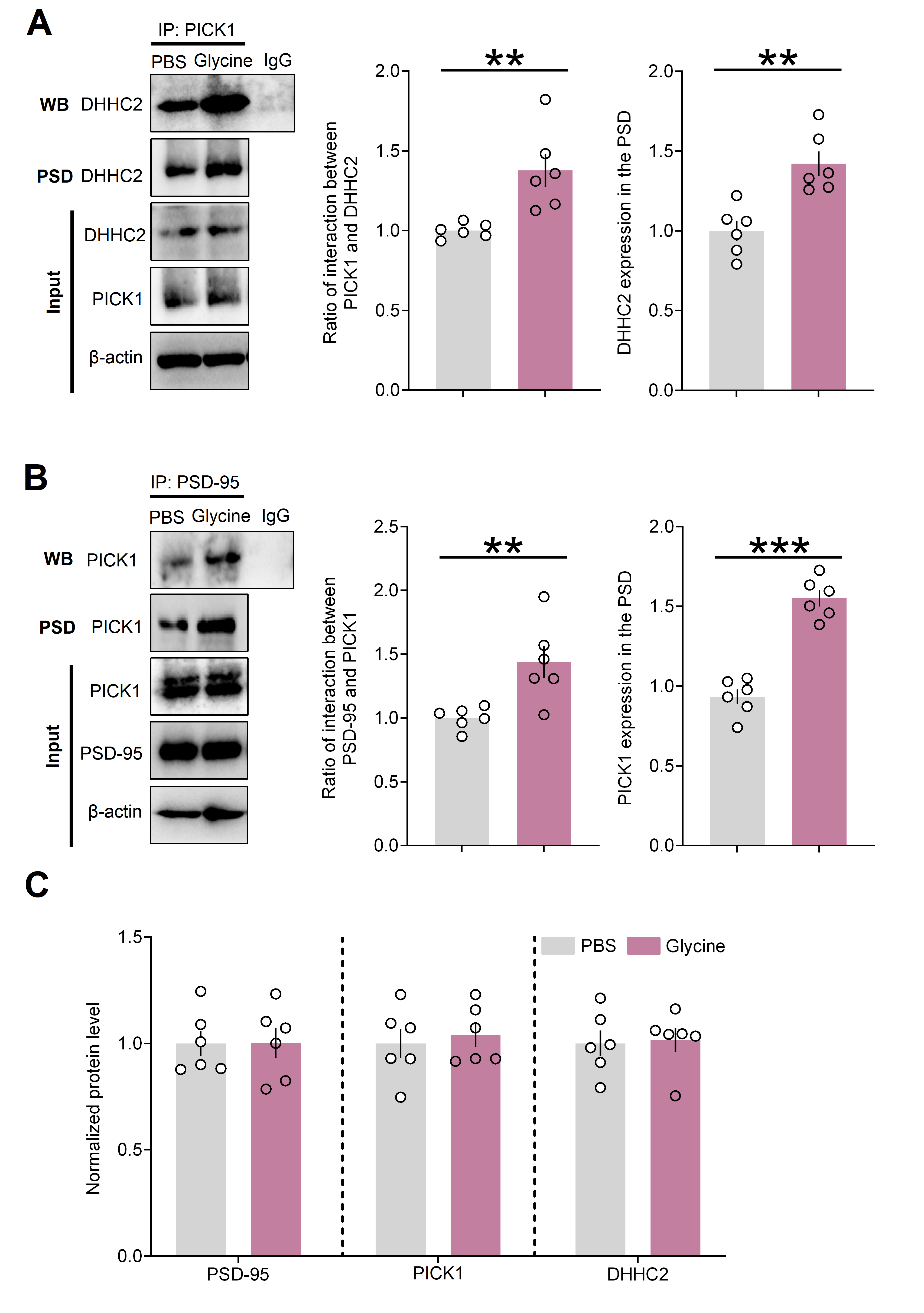
**

**Figure S10. Glycine mediates the interaction between PICK1 and DHHC2.** (**A**) Representative Western blot results (left) and quantitative analysis (right) showing the expression levels of PICK1 and DHHC2 in PSD-enriched fractions, as well as their interaction (detected via Co-IP) in PBS-treated and glycine-treated hippocampal neurons. (**B**) Representative Western blot results (left) and quantitative analysis (right) showing the expression levels of PICK1 and PSD-95 in PSD-enriched fractions, as well as their interaction (detected via Co-IP) in PBS-treated and glycine-treated hippocampal neurons. (**C**) Quantitative analysis of PICK1, DHHC2, and PSD-95 expression levels in total lysates (n = 6 rats per group). Data are represented as mean ± SEM. **p < 0.01, ***p < 0.001. Statistical analyses were performed using one-way ANOVA followed by Tukey’ s post hoc test.

**Supplementary Tables**

**Supplementary Table 1. Information of antibodies used in Western blot assays.**

| **Anti-protein** | **Dilution** | **Host** | **Band size** | **Catalog** | **Manufacturer** |
| --- | --- | --- | --- | --- | --- |
| GluA1 | 1/1000 | Rabbit | 100 kDa | ab31232 | Abcam, UK |
| GluA2 | 1/1000 | Rabbit | 100 kDa | ab20673 | Abcam, UK |
| GluA3 | 1/1000 | Rabbit | 100 kDa | ab40845 | Abcam, UK |
| GluA4 | 1/1000 | Rabbit | 100 kDa | ab53088 | Abcam, UK |
| PSD-95 | 1/1000 | Rabbit | 95 kDa | #3409 | CST, USA |
| PICK1 | 1/1000 | Mouse | 47 kDa | 10983-2-AP | Proteintech, China |
| DHHC2 | 1/500 | Rabbit | 42 kDa | DF4688 | Affinity, USA |
| DHHC3 | 1/1000 | Rabbit | 34 kDa | ab31837 | Abcam, UK |
| DHHC5 | 1/1000 | Rabbit | 78 kDa | ab200572 | Abcam, UK |
| DHHC7 | 1/1000 | Rabbit | 35 kDa | ab138210 | Abcam, UK |
| DHHC8 | 1/500 | Mouse | 81 kDa | sc-374191 | Santa Cruz, USA |
| DHHC15 | 1/500 | Rabbit | 39 kDa | orb451302 | Biorbyt, UK |
| APT1 | 1/500 | Rabbit | 25 kDa | ab91606 | Abcam, UK |
| β-actin | 1/3000 | Mouse | 43kDa | sc-47778 | Santa Cruz, USA |
| HRP-Streptavidin | 1/1000 |  |  | BA1088 | BOSTER, China |
| Donkey anti-rabbit Alexa Fluor 594 | 1/1000 | Secondary Antibody | | #A-21207 | Thermofisher Scientific |
| Donkey anti-rabbit Alexa Fluor 488 | 1/1000 | Secondary Antibody | | #A-21206 | Thermofisher Scientific |
| Donkey anti-mouse Alexa Fluor 594 | 1/1000 | Secondary Antibody | | #A-11032 | Thermofisher Scientific |
| Donkey anti-mouse Alexa Fluor 488 | 1/1000 | Secondary Antibody | | #A-11029 | Thermofisher Scientific |
| Goat anti-Mouse IgG (H+L) | 1/10000 | Secondary Antibody | | #31430 | Thermofisher Scientific |
| Goat anti-Rabbit IgG (H+L) | 1/10000 | Secondary Antibody | | #31460 | Thermofisher Scientific |
| Mouse anti-Goat IgG (H+L) | 1/10000 | Secondary Antibody | | #31400 | Thermofisher Scientific |

**Supplementary Table 2. Oligonucleotide primers used for mRNA real time PCR.**

| **Gene** | **Sense primer (5’ to 3’)** | **Antisense primer (5’ to 3’)** |
| --- | --- | --- |
| DHHC2 | AGAGCCAAGAGGAGAAGCCCATC | AGTCGGCATCTGTCACAATATCGG |
| ABHD17B | GGACCGTACC GTCTGTGGAC | CCGCATTCCTGAGGTCAAAG |
| GAPDH | ATGGTGAAGGTCGGTGTG | CATTCTCGGCCTTGACTG |

**Supplementary Table 3. Binding affinity and K_D_ prediction between DHHC2 and PICK1.**

| **Protein-protein complex** | **△G (kcal/mol)** | **K_d_ (M) at ℃** | **ICs charged-charged** | **ICs charged-polar** | **ICs charged-apolar** | **ICs polar-polar** | **ICs polar-apolar** | **ICs apolar-apolar** | **NIS charged** | **NIS apolar** |
| --- | --- | --- | --- | --- | --- | --- | --- | --- | --- | --- |
| Combine | -12.2 | 1.2e-09 | 0.0 | 3.0 | 12.0 | 5.0 | 31.0 | 27.0 | 19.95 | 44.3 |
